# Role of Fe decoration on the oxygen evolving state of Co_3_O_4_ nanocatalysts†

**DOI:** 10.1039/d3ee02809g

**Published:** 2024-01-30

**Authors:** Felix T. Haase, Eduardo Ortega, Sascha Saddeler, Franz-Philipp Schmidt, Daniel Cruz, Fabian Scholten, Martina Rüscher, Andrea Martini, Hyo Sang Jeon, Antonia Herzog, Uta Hejral, Earl M. Davis, Janis Timoshenko, Axel Knop-Gericke, Thomas Lunkenbein, Stephan Schulz, Arno Bergmann, Beatriz Roldan Cuenya

**Affiliations:** a Department of Interface Science, Fritz Haber Institute of the Max Planck Society Berlin Germany abergmann@fhi-berlin.mpg.de roldan@fhi-berlin.mpg.de; b Department of Inorganic Chemistry, Fritz Haber Institute of the Max Planck Society Berlin Germany; c Institute for Inorganic Chemistry and Center for Nanointegration Duisburg-Essen [CENIDE], University of Duisburg-Essen Essen Germany; d Max-Planck-Institut für Chemische Energiekonversion, Stiftstrasse 34-36 45470 Mülheim Germany

## Abstract

The production of green hydrogen through alkaline water electrolysis is the key technology for the future carbon-neutral industry. Nanocrystalline Co_3_O_4_ catalysts are highly promising electrocatalysts for the oxygen evolution reaction and their activity strongly benefits from Fe surface decoration. However, limited knowledge of decisive catalyst motifs at the atomic level during oxygen evolution prevents their knowledge-driven optimization. Here, we employ a variety of *operando* spectroscopic methods to unveil how Fe decoration increases the catalytic activity of Co_3_O_4_ nanocatalysts as well as steer the (near-surface) active state formation. Our study shows a link of the termination-dependent Fe decoration to the activity enhancement and a significantly stronger Co_3_O_4_ near-surface (structural) adaptation under the reaction conditions. The near-surface Fe– and Co–O species accumulate an oxidative charge and undergo a reversible bond contraction during the catalytic process. Moreover, our work demonstrates the importance of low coordination surface sites on the Co_3_O_4_ host to ensure an efficient Fe-induced activity enhancement, providing another puzzle piece to facilitate optimized catalyst design.

## Abstract 2

Electrocatalytic OER activity of Co_3_O_4_ is enhanced by Fe near-surface decoration with a more pronounced formation of Co–FeO_*x*_(OH)_*y*_ reaction zones comprising distinct redox-active Fe^3+^ and Co^2+^ sites as shown by comprehensive *operando* studies.

## Graphical abstract

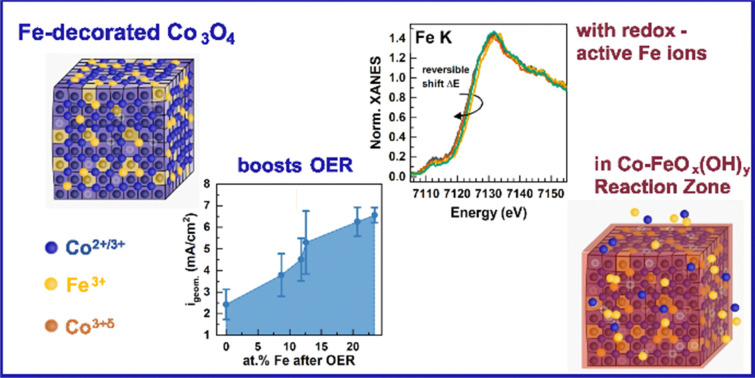

**Broader context**

Environmentally friendly technologies to convert electrical energy from renewable power sources and produce hydrogen are essential to facilitate the industrial and societal transformation towards carbon neutrality. Here, alkaline water electrolysis can play a major role but, currently, the efficiency of green hydrogen production is largely limited by the overpotential of the anodic oxygen evolution reaction (OER) and thus, more active and stable electrocatalysts are needed. For the alkaline OER, 3d transition metal oxide electrocatalysts such as Co_3_O_4_ and Ni(OH)_2_ are usually applied as electrocatalysts and it has been shown that the catalytic activity can be boosted by Fe addition. However, it is still unclear how the Fe interacts with the surface of the (crystalline) host catalyst and how it steers the formation of the oxygen-evolving catalyst state and thus increases the catalytic activity. This information is essential to facilitate the knowledge-driven catalyst design for large scale H_2_ production. Herein, we studied a series of Co_3_O_4_ nanocatalysts that we decorated with Fe electrolyte species in a controlled way to understand the structural, compositional and morphological roles of Fe on the catalytic OER activity and the active state formation. We were able to link the preferred Fe location to the shape of the Co_3_O_4_ nanocatalyst and the increase in OER activity. By using a variety of advanced bulk- and surface-sensitive operando methods, we revealed the essential differences in the oxygen-evolving state of Fe-decorated and Fe-free Co_3_O_4_ providing an important puzzle piece for a comprehensive understanding of the OER. Our results also highlight the necessity to consider the nanoscale heterogeneity of industrially relevant electrocatalysts to understand and design high-performing materials for the alkaline water electrolysis.

## Introduction

Hydrogen is regarded as the key feedstock and fuel in the carbon emission-free industry making its production an integral step towards clean energy conversion. It is therefore crucial to promote the production and application of so-called green hydrogen obtained from water electrolysis. During water electrolysis, the anodic oxygen evolution reaction (OER) is regarded as the bottleneck because of the sluggish kinetics involved in the 4-electron mechanism.^1,2^ Crystalline Co_3_O_4_ spinel catalysts are promising low-cost and stable anode materials for alkaline water electrolysis with high OER activity and stability.^3–8^ In addition, Co_3_O_4_-based electrocatalysts have shown superior performance in anion exchange membrane electrolyzers compared to liquid electrolyte state-of-the-art NiFeO_*x*_ catalysts due to their better conductivity and suppressed degradation.^9^

An elegant approach to enhance the OER performance of Co- and Ni-oxides, demonstrated by Corrigan,^10,11^ is to employ surface Fe decoration, *e.g.* from electrolyte impurities. Although this finding has attracted substantial attention in the last decade,^12–15^ the current state of knowledge does not provide a coherent picture of the catalytic role and active state properties of the Fe sites in the OER. Generating a unified picture of the catalytic role of Fe would enable a purely knowledge-driven catalyst optimization. On the one hand, it has been shown that the Fe^3+^ ions incorporated into MOOH (M = Co, Ni) matrices remain in a 3+ valence state^16,17^ and enhance the transition of Ni^2+^ to Ni^4+^ sites.^18,19^ On the other hand, it was shown that Fe^3+^ gets oxidized during the OER,^20–22^ which supposedly hampers the oxidation of Ni^2+^ to Ni^3+^ sites.^23^ Additionally, Fe may also benefit from a stabilizing, conductive host^17,24,25^ or might be found embedded in the crystalline structure of the OER active site.^16,26^ Thus, the chemical state and electronic structure of the oxygen-evolving Fe-containing near-surface is unclear. In this regard, it is important to note that revealing the oxidation of a transition metal M (=Co, Ni, Fe) with a valence state beyond M^3+^ requires careful analysis, as simultaneously electrophilic oxygen species, potentially crucial for the O–O bond formation, can form.^15,21,27,28^

So far, the interaction of solvated Fe ions with the surface of Co-based oxides has been studied mostly on amorphous CoO_*x*_(OH)_*y*_ materials and the interaction with crystalline Co_3_O_4_ catalysts remains relatively unknown. Specifically, the reversible formation of the CoO_*x*_(OH)_*y*_ adaption layer on Co_3_O_4_ during the OER^8,29^ could facilitate Fe incorporation into the near-surface spinel lattice, thus leading to OER activity enhancement.^30–34^ In previous studies on Co_3−*x*_Fe_*x*_O_4_ spinels, we correlated higher OER activity with a more pronounced Co oxidation and CoO_*x*_(OH)_*y*_ adaption layer formation with a Co : Fe surface ratio of 3 : 1.^35,36^ For epitaxial Co_3−*x*_Fe_*x*_O_4_(111) thin films, Fe-containing Co_1+*δ*_Fe_2−*δ*_O_4_(111) are more active than the bare Co_3_O_4_(111), and evolve towards the Co-richer surface termination upon the OER.^37^ However, the location as well as the electronic structure and chemical state of the Fe species interacting with the Co_3_O_4_ host remain ambiguous. Identifying the preferred (most stable) Fe decoration sites on Co_3_O_4_ as well as their (electronic) structure during oxygen evolution is critical to generate a unified picture of the Co–Fe catalytically active surface.

In this study, we tracked the incorporation of cationic Fe species from the electrolyte onto Co_3_O_4_ nanostructures. After Fe deposition, we found an affinity of Fe with Co_3_O_4_(100) surface sites using scanning transmission electron microscopy (STEM) and energy dispersive X-ray (EDX) mapping. Moreover, our findings suggest a saturating Fe concentration on specific surface sites, which we can correlate with the OER enhancement. Surprisingly, our *operando* investigations show subtle differences in the Co chemical state between the Fe-free and Fe-containing active catalyst's states. In the latter case, we identified combined accumulation of oxidative charges at the Fe, Co, and O sites during the OER, suggesting that the Fe–O also participates in redox electrochemistry as well as OER catalysis. Thus, our study provides new insights into the nature of Fe deposition on Co_3_O_4_ sites and the OER-active Fe–O sites.

## Results and discussion

**Fig. 1:**
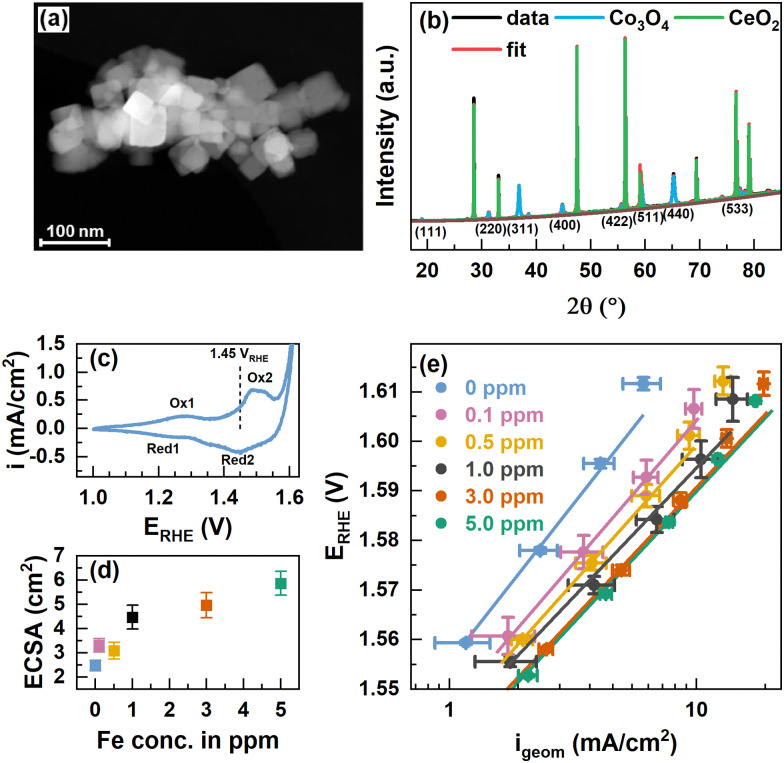
(a) Scanning transmission electron microscopy (STEM) annular dark-field (ADF) image of Co_3_O_4_ NPs. The scale bar corresponds to 100 nm. (b) XRD patterns of Co_3_O_4_ NPs and CeO_2_ NIST SRM674b standard with fits from Rietveld refinement. The fit parameters are shown in Table S1 (ESI†). (c) Cyclic voltammograms with 100 mV s^−1^ in purified 0.1 M KOH and indicated oxidation and reduction features. (d) Electrochemically active surface area (ECSA) extracted from cyclic voltammograms in the range of 0.95–1.05 V_RHE_ in Fe-free 0.1 M KOH with different scan rates and linear regression of cathodic and anodic charging currents. The measured capacitance was normalized using a specific capacitance of 0.040 mF cm^−2^ which is suggested for metal oxides.^42^ Error bars reflect the standard deviations from 3 individual measurements. (e) Tafel plots from potential step experiments in Fe-free 0.1 M KOH with 20 mV potential steps applied for 2 minutes each. Measurements were performed after conditioning at 1.45 V_RHE_ for 1 h in Fe-free, and 0.1, 0.5, 1.0, 3.0, and 5.0 ppm Fe-containing 0.1 M KOH. Tafel slopes are 65.0 ± 0.3, 57.4 ± 0.6, 55.6 ± 0.4, 52 ± 3, 53.8 ± 0.9 and 53 ± 1 mV dec^−1^, respectively.

Co_3_O_4_ spinel oxide nanoparticle (NP) catalysts were synthesized following a solvothermal route from Co(NO_3_)_2_ using oleylamine as the capping agent and consecutive calcination at 300 °C.^38^ The X-ray photoelectron spectroscopy (XPS) data of the O and C1s peak regions showed the removal of organic ligands, Fig. S1 (ESI†). Fig. 1(a) shows a conglomerate of well-defined cubically shaped Co_3_O_4_ NPs alongside smaller, rather spherical non-cubic NPs. The particle sizes range between 15 and 50 nm and on average of 20–30 nm, see also Fig. S2 (ESI†). The cubic Co_3_O_4_ NPs expose primarily a (100) termination, while the rounded corners and non-cubic NPs derive likely from (110) and (111) and higher indexed orientations.^38–40^

We recorded powder X-ray diffractograms of Co_3_O_4_ which confirm the phase purity, and we determined a total crystallinity of 85.8% by using a CeO_2_ reference (NIST SRM674b), Fig. 1(b). The Bragg peaks solely correspond to the Co_3_O_4_ spinel as well as the CeO_2_ and using Rietveld refinement we determined a volume-averaged crystallite size of ∼32 nm and a lattice parameter of ∼8.087 Å which agrees well with Co_3_O_4_, Table S1 (ESI†). The surface chemical state of the as-prepared Co_3_O_4_ NPs agrees well in the presence of a mixed Co^2+/3+^ valence state as the Co 2p XPS regions shown in Fig. S3 (ESI†) strongly resemble that of Co_3_O_4_ presented in the literature.^29,41^ Depth-dependent XPS analysis using kinetic energies of photoelectrons of ∼550 eV and ∼200 eV corresponding to information depths of ∼10 and ∼5 Å, respectively, suggest a Co^2+^-richer termination layer due to the slightly larger Co^2+^ satellite contribution as denoted in Table S2 (ESI†).

To vary the degree of the Fe surface decoration of the Co_3_O_4_ NPs, cationic Fe was anodically deposited at 1.45 V_RHE_ for 1 h from Fe^3+^(NO_3_)_3_ in Fe-purified 0.1 M KOH. The potential of 1.45 V_RHE_ was chosen to reflect non-catalytic conditions below the OER onset potential (∼1.55 V_RHE_) and between the two major Co oxide redox transitions – Co^2+^/Co^3+^ oxidation at 1.25 V_RHE_ and the Co^3+^/Co^3+*δ*^ oxidation at 1.52 V_RHE_, Fig. 1(c). Thereby, we aimed to ensure the morphological integrity of the NCs in addition to facilitating the interaction of the deposited Fe species with the formed CoO_*x*_(OH)_*y*_ surface structure and thus to the formation of an Fe-containing Co_3_O_4_ surface and potentially subsurface regions. The Fe is expected to be deposited on the Co^3+^–O surface sites, *i.e.* the sites formed from the initially tetrahedrally coordinated Co^2+^ sites of the Co_3_O_4_ near-surface. Upon conditioning, the electrochemical reduction of the Fe-containing CoO_*x*_(OH)_*y*_ near-surface will lead to the incorporation of the Fe species into the near-surface spinel lattice. We have to note the possibility that the selective oxidation of Co^2+^ sites could induce a selective deposition of Fe on Co_3_O_4_, contrasting Fe deposition on the more strongly oxidized CoO_*x*_(OH)_*y*_ at higher electrode potentials during the OER. The Fe content in the electrolyte was varied between 0 and 5 ppm Fe and two exemplary chronoamperometry profiles of the conditioning with and without Fe are shown in Fig. S4 (ESI†). Measurements in the Fe-purified electrolyte after Fe conditioning show an increase of the electrochemically active surface area (ECSA) following the Fe concentration in the electrolyte, Fig. 1(d).

Revisiting the Pourbaix diagram of Fe and earlier works shows the instability of Fe oxides under OER conditions^43^ and thus, we studied the irreversible changes in the structure, morphology and composition of our materials after the catalytic testing in the Fe-free electrolyte. To quantify the catalytic improvement introduced by the Fe decoration of the surface of the CO_3_O_4_ NPs, we performed potential step experiments and Tafel analysis, Fig. 1(e). Overall, the current density in the whole potential regime increases with the Fe concentration and shows a ∼3-fold increase at 1.58 V from ∼2 mA cm^−2^ for the Fe-free Co_3_O_4_ to ∼6 mA cm^−2^ for the Co_3_O_4_ conditioned in 5 ppm Fe. Although there is a substantial increase in OER activity, it is apparently less pronounced for the crystalline Co_3_O_4_ NPs compared to electrodeposited CoO_*x*_(OH)_*y*_ catalysts.^17,43^ Notably, the current density of the NPs is not stable at higher potentials and the current decrease is more pronounced for lower Fe concentrations, which suggests an OER activity-stabilizing effect of the Fe decoration. The Tafel slopes decrease with increasing Fe concentrations until a minimum of 52–54 mV dec^−1^ is reached for ≥1 ppm Fe suggesting the enhanced OER kinetics in the Fe-decorated case. Notably, the Fe conditioning does not have a pronounced influence on the Co-related redox transitions, Fig. S5 (ESI†). This finding is in agreement with earlier works on Co_3−*x*_Fe_*x*_O_4_^44,45^ but contrasts the previously seen pronounced anodic shift with Fe incorporation into Co/Ni-O_*x*_(OH)_*y*_ catalysts.^17,46^

**Fig. 2:**
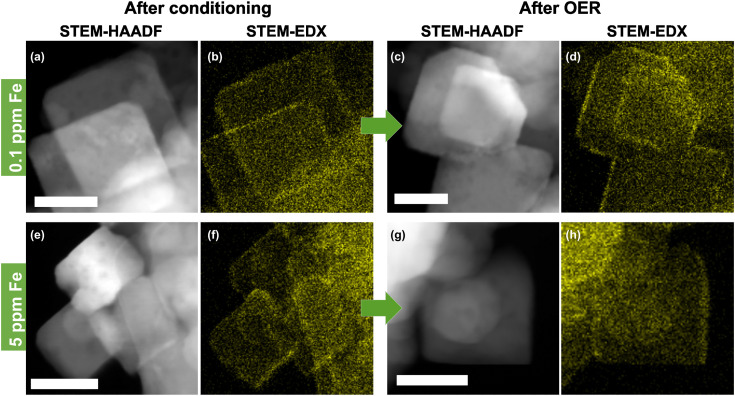
Scanning transmission electron microscopy-high angle annular dark-field (STEM-HAADF) images and corresponding energy dispersive X-ray spectroscopy (EDX) Fe-K_α_ color maps of Co_3_O_4_ catalysts after conditioning for 1 h at 1.45 V_RHE_ in 0.1 M KOH and after the OER in Fe-purified 0.1 M KOH for 30 min at 1.6 V_RHE_. (a) and (b) Conditioning with 0.1 ppm Fe and (c) and (d) after the OER. (e) and (f) Conditioning with 5.0 ppm Fe and (g) and (h) after the oxygen evolution reaction (OER). Further images of all samples are shown in Fig. S6–S9 (ESI†). The scale bar is 20 nm.

The presence of well-defined cubic as well as non-cubic NPs allows the investigation of the preferred location for Fe decoration on Co_3_O_4_ both before and after catalysis in the Fe-purified electrolyte. Thus, STEM-high angle annular dark-field (STEM-HAADF) with EDX mapping was performed, Fig. 2 and Fig. S6–S9 (ESI†). This shows that the conditioning with and without the addition of Fe as well as the subsequent OER catalysis do not alter the morphology of the NPs substantially. Only a slight increase in the roughness of the NP surface compared to the as-prepared state can be seen. High resolution (HR)-STEM measurements of the NPs allowed resolving Fe moieties on the surface after conditioning in Fe-free and 5.0 ppm Fe electrolytes, Fig. S10 and S11 (ESI†). In both cases, the cubic NPs retain their morphology exposing (100) facets and neither on (100) nor on (110)/(111) facets were we able to resolve FeO_*x*_ NP formation. By tilting individual cubically shaped NPs and performing EDX line scans, we show that Fe is distributed on the (100) facets, Fig. S12 and S13 (ESI†). In particular, this analysis shows that the Fe accumulates in the upper ∼2 nm of Co_3_O_4_ and does not penetrate into the bulk of the nanocubes. Thus, we can conclude that the Fe decoration leads to the formation of a thin layer of Fe-containing Co_3_O_4_. Similar findings were reported previously while studying the interaction of Fe impurities in the electrolyte with Ni oxide NPs during the OER. In particular, using EC-TEM, we unveiled the initial formation of a NiFe-LDH skin layer.^47^ Nonetheless, further electrochemical cycling in the presence of Fe did not lead to additional Fe incorporation into the subsurface/bulk of the NiO, but to its accumulation on the surface as Fe oxide NPs. In the case of the Fe-conditioned Co_3_O_4_, STEM-EDX mapping after the OER in the Fe-purified electrolyte at 1.6 V_RHE_ for 30 min proves the stability of the Fe decoration under OER conditions, Fig. 2 and Fig. S8 and S9 (ESI†). Additionally, we cannot rule out the partial irreversible formation of hydroxylated Fe/Co sites in the near-surface exclusively based on the *ex situ* spectro-microscopy analysis conducted here (TEM-EDX). Recently, we have shown using quasi *in situ* XPS that the Co_3_O_4_ surface appears hydroxylated after an extended exposure to OER conditions and that the OH/Lattice-O ratio is equivalent to a sub-nm hydroxide film. These measurements were however carried out after the OER, a sample rinse in water, and a subsequent sample transfer to the analysis chamber without air exposure.^37^

**Fig. 3:**
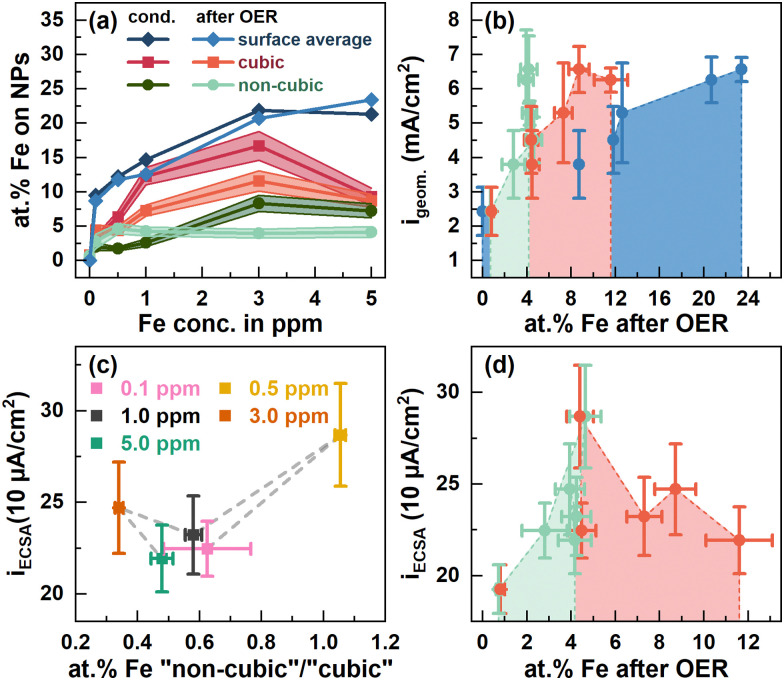
(a) at% Fe at the surface of cubic (red) and non-cubic Co_3_O_4_ NPs (green) from STEM-EDX measurements, Fig. S6–S9 (ESI†), Tables S3 and S4 (ESI†), and the sample averaged at% Fe from X-ray photoelectron spectroscopy (blue) after conditioning in different ppm Fe and after OER measurements displayed in Fig. 2. ECSA-normalized specific current densities at 1.58 V_RHE_ from Fig. 1 dependent on (c) the at% Fe at non-cubic/cubic site ratios after the OER and (d) the cubic and non-cubic at% Fe concentrations.

To quantify the surface Fe concentration before and after the OER on the Co_3_O_4_ NPs, we determined the Co and Fe EDX signals at the edges and the center of the cubic and the rounded particles, Fig. S6–S9 (ESI†), and performed the XPS analysis to reveal the changes in the chemical state and the average Fe : Co ratio on the surface. Notably, at the energies employed, the probing volume of STEM-based EDX (thickness of ∼4 nm) is substantially larger than that of XPS (thickness of 1 nm at 550 eV kinetic energy) and thus, the obtained data cannot be compared on a quantitative basis. Thus, the lower Fe/Co ratios obtained by STEM-EDX simply reveal less Fe-rich (or Fe-absent) regions in the core of the cobalt oxide particles that are not probed *via* the more surface-sensitive XPS analysis. We found that the surface-average Fe : Co ratio increases with the Fe concentration during the conditioning from 9.5 at% at 0.1 ppm to ∼21 at% Fe for 3 and 5 ppm Fe, Fig. 3(a). Independent of the information depth, the XPS profiles after the OER resemble the same Co spinel features, with primarily Fe^3+^ and a similar Fe : Co ratio as that measured after the 5 ppm conditioning, Fig. S15 (ESI†). Therefore, the NP surface remains Co_3_O_4_-like after the OER, and the cationic Fe species incorporated into the top layers of the spinel NP. Depth-dependent compositional analysis shows 22 at% Fe and 27 at% for 1 nm and 0.5 nm information depths, respectively, upon conditioning, Fig. S14 (ESI†). Notably, this depth analysis does not exceed the thickness of the Fe-containing layer determined by STEM-EDX as shown in Fig. S12 (ESI†). Therefore, it is understandable that the depth-dependent compositional analysis data obtained from XPS do not vary strongly between 0.5 and 1 nm probe depths. Thus, we can use the TEM-EDX data to conclude that the Fe did not migrate into the bulk of the Co_3_O_4_ NPs after conditioning, but remained in the top surface layers.

After the OER, the sample-averaged Fe : Co ratio exhibits only a small decrease of up to 1 at% Fe, which suggests a generally stable Fe configuration. Independent of the information depth, the XPS profiles after the OER resemble the same Co spinel features with primarily Fe^3+^ and a similar Fe : Co ratio after the 5 ppm conditioning, Fig. S15 (ESI†). Therefore, the NP surface remains Co_3_O_4_-like after the OER with the cationic Fe species incorporated into the near-surface.

The semi-quantitative EDX analysis confirms that after conditioning at lower Fe concentrations the Fe accumulates preferably on the (100) facets. In the regime from 0.1 to 1 ppm Fe, the Fe : Co ratio increases from 3 to 12 at%, while almost no Fe is found on the near-surface of non-cubic particles (2–2.6 at% Fe). On the (100) facets, the highest Fe : Co ratio (16.7 at%) is present after the 3 ppm conditioning but the Fe : Co ratio is lower (9.3 at%) after 5 ppm conditioning. On the non-cubic NPs, we identified a qualitatively identical behavior though with a lower Fe : Co ratio of maximally 8 at% Fe.

Upon the OER, the Fe : Co ratios on the near-surface of cubic NPs decreased substantially, except for the Co_3_O_4_ conditioned with 5 ppm Fe. Concurrently, the Fe : Co ratio on the non-cubic NPs converged to 4 at%, independent of the initial Fe : Co ratio thereon and thus, the Fe : Co ratio on the non-cubic NP increased for the Co_3_O_4_ conditioned with 1 ppm Fe and below. Since the sample-averaged Fe ions did not leach from the Co_3_O_4_ near-surface as shown by XPS, the Fe ions partially rearranged within the NP ensemble upon the OER in the Fe-free electrolyte. This process is in agreement with the discussed dissolution–redeposition cycles of Fe (and Co) ions from and to the CoO_*x*_(OH)_*y*_ reaction zone.^25^ Furthermore, the rearrangement suggests that the tendency of Fe deposition under OER conditions shifts slightly towards the non-cubic NPs with a most stable Fe : Co ratio of 4 at% as compared to the cubic NPs. However, the cubic NPs still seem to be preferred for the Fe incorporation as its Fe : Co ratio is higher also after the OER. Notably, the obtained Fe : Co ratio after the OER agrees well with the obtained optimum for the highest catalytic activity as it has been reported for Fe–metal oxyhydroxides.^25^ Intriguingly, values of 3 : 1 and 3.5 : 1 have been reported as the optimal Fe : Co ratios for OER activity in mixed Co_3−*x*_Fe_*x*_O_4_ spinel catalysts.^35,36,48^

Evidently, there is a clear influence of the Fe decoration on the catalytic activity and here, different interactions of the Fe with cubic and non-cubic NPs are expected to play a crucial role. Thus, we linked the apparent and specific OER activity *via* the geometric and ECSA-normalized current density at 1.58 V_RHE_ to the surface-average Fe : Co ratio as well as more specifically the Fe content on the non-cubic to cubic NPs after the OER, Fig. 3(b)–(d). The apparent OER activity monotonously increases with the surface-average Fe ratio, Fig. 3(b). This behavior can be linked to the increase in the electrochemical surface area and thus, likely the number of active sites with the increasing surface-averaged Fe : Co ratio, Fig. 1d. Similarly, the apparent OER activity increases with the Fe content on the non-cubic and cubic NPs up to ∼4 and 8 at%, respectively, Fig. 3(b). In contrast, the specific OER activity is with 287 μA cm^−2^ highest for ∼4 at% Fe on the non-cubic and cubic NPs after the OER, Fig. 3(c) and (d). For higher Fe contents and thus, more pronounced decoration of the cubic NPs, a specific activity of ∼220–250 μA cm^−2^ irrespective of the Fe content is only slightly higher compared to that of the Fe-free Co_3_O_4_ (∼200 μA cm^−2^). Thus, we conclude a larger fraction of Fe on non-cubic NPs after the OER enhances the specific OER activity stronger compared to Fe sites on the cubic NPs, Fig. 3(c). Overall, the Fe-induced enhancement of the apparent OER activity can be mostly attributed to an Fe-induced increase of the electrochemical surface area. However, we unveiled that the addition of Fe enhances the specific OER activity of the Co_3_O_4_ catalyst only for low Fe amounts and rather on non-cubic NPs. The Fe adsorption affinity remains facet- and morphology-dependent on Co_3_O_4_, which turns out to be the limiting characteristic for further OER-activity increases. It is unclear whether a stronger Fe decoration of non-cubic NPs with its apparently higher specific activity would lead to a stronger enhancement of the apparent OER activity. Furthermore, we expect that higher overall Fe decoration could lead to decreasing OER activity following the volcano like activity profile of the Fe : Co ratio.^17,35,36,43^

To fully understand the catalytic role of the cationic Fe species, the properties of the working catalysts have been identified. On the crystalline Co_3_O_4_, the formation of an X-ray amorphous CoO_*x*_(OH)_*y*_ skin-layer or the reaction zone during the OER is well known,^8,29,49,50^ but active state properties in the presence of Fe surface decoration are not yet fully understood. We therefore applied a variety of bulk- and surface-sensitive *operando* methods to gain comprehensive insight into the Fe-free and Fe-decorated Co_3_O_4_ NPs during the OER. Prior to the OER measurements in the Fe-free electrolyte, all catalysts were conditioned in either 5 ppm Fe-containing 0.1 M KOH or the same electrolyte without Fe. Subsequently, the OER active state was probed by chronoamperometry for 30 minutes at 1.6 V_RHE_ and measurements after the OER at 1 V_RHE_.

**Fig. 4:**
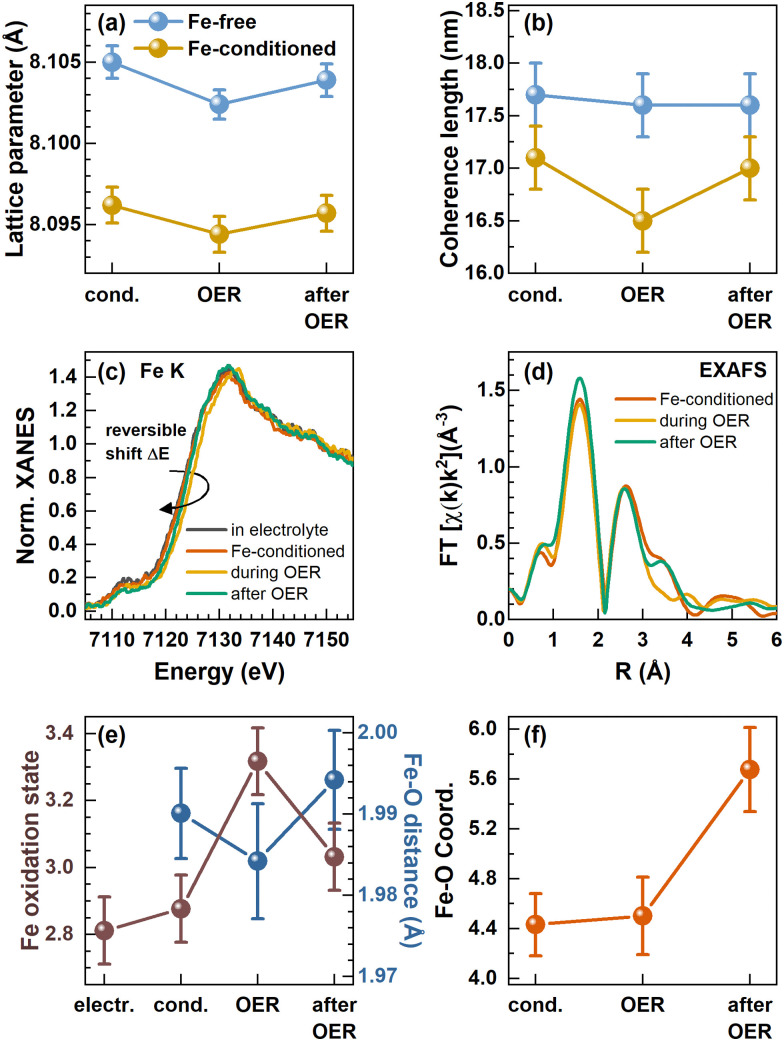
*In situ* and *operando* measurements in a Fe-purified electrolyte and after conditioning with Fe. (a) Lattice parameter and (b) coherence length of the Co_3_O_4_ spinel retrieved from Rietveld refinement of *in situ* high-energy X-ray diffraction. (c) *Operando* XANES at the Fe K-edge with an indicated reversible shift of the absorption edge to higher binding energies during the OER and (d) *operando* Fourier transformed (FT) X-ray absorption fine structure (FT-EXAFS). (e) Correlation of the Fe oxidation state calculated from the linear regression of Fe reference absorption edge positions and the Fe–O bond distance from EXAFS fitting. (f) Fe–O coordination number evolution from fitting the first Fe–O coordination shell visible in FT-EXAFS. The error bars for the coordination numbers and Fe–O distances are provided by the standard EXAFS fitting procedure and the error bars for the Fe oxidation state were calculated from an energy resolution of 0.1 eV and the integration of the absorption edge.

We followed the structural transformation of the Co_3_O_4_ and the CoO_*x*_(OH)_*y*_ reaction zone formation by *operando* high-energy XRD for Fe-free and 5 ppm Fe conditioned catalysts, Fig. 4(a) and (b). Rietveld refinement showed that the Co_3_O_4_ spinel adapts to the OER conditions as the lattice contracts reversibly during the OER, which has been previously attributed to strain at the Co_3_O_4_–CoO_*x*_(OH)_*y*_ interface.^8,29,49,50^ Under the Fe-free conditions, we did not detect any pronounced change in the Co_3_O_4_ coherence length during the OER, likely due to the comparably large domain size limiting the sensitivity of XRD. However, the electrochemical conditioning in the presence of Fe leads to an irreversible decrease of the Co_3_O_4_ lattice parameter as well as the structural coherence length under non-catalytic conditions. This change can be explained based on the irreversible formation of the Co_3−*x*_Fe_*x*_O_*x*_(OH)_*y*_ near-surface region during the Fe conditioning which deviates at least in the outermost 0.5 nm structurally from the Co_3_O_4_ bulk of the NPs. We also emphasize the agreement between the lower structural coherence length and the increased electrochemical surface area, which suggests a smaller crystallite/particle size. However, we did not detect a significant decrease in the size of the cubic or non-cubic Co_3_O_4_ NPs upon Fe conditioning. Thus, the Co_3−*x*_Fe_*x*_O_*x*_(OH)_*y*_ near-surface region is likely solely responsible for the increase in the ECSA.

Importantly, we show that the coherence length of the Fe-decorated Co_3_O_4_ clearly and reversibly decreases during the OER. This finding indicates the more pronounced formation of a Co_3−*x*_Fe_*x*_O_*x*_(OH)_*y*_ near-surface layer, which agrees well with the obtained link between the catalytic activity and the formation of an oxyhydroxide reaction zone.

We did not see the formation of new phases besides the spinel phase like CoO_*x*_(OH)_*y*_-related Bragg peaks, Fig. S16 (ESI†), suggesting that only the near-surface of the NPs transform, which likely comprise the active sites for the OER. We have to emphasize here that this reversible transformation does not exclude a partial, irreversible hydroxylation of the (Fe-containing) Co_3_O_4_ near-surface after the reaction, as it has been found for Co_3−*x*_Fe_*x*_O_4_ thin film model surfaces after exposure to OER conditions.^37^

To resolve the changes in the local structure and oxidation state during the active state formation, we complementarily employed *operando* metal K-edge X-ray absorption spectroscopy (XAS).^16,28,29,51^ Similar to the *operando* HE-XRD insights, Co K-edge XAS cannot resolve the changes of the NP near-surface under OER conditions likely due to the low surface–volume ratio of the relatively large 15–50 nm Co_3_O_4_ catalysts, Fig. S17 (ESI†). Under all conditions investigated, the Co K-edge X-ray absorption near edge structure (XANES) thus clearly resembles that of the Co_3_O_4_ spinel.

In contrast, the Fe K-edge XANES spectra recorded for the Fe-conditioned Co_3_O_4_ show clear changes under different electrochemical conditions, Fig. 4(c), and the overall shape of the absorption edge clearly resembles the one of the Fe^3+^ references, Fig. S18 (ESI†). However, the Fe K-edge position shifts reversibly to higher energies during the OER, which suggests a reversible oxidation of the Fe–O species in the working catalyst. Following the calibration of the edge shift with the formal Fe oxidation state, we determined a formal Fe oxidation state during the OER of ∼+3.3. In addition to this shift, the Fe K pre-edge feature at ∼7112 eV decreases during the OER and further after the OER, which suggests irreversible changes in the Fe–O coordination symmetry towards octahedral during catalysis.^52,53^ We followed the Fe content under electrochemical conditions by determining the edge step of the non-normalized Fe K-edge XANES which increases during the conditioning while it remains constant during the OER in the Fe-free electrolyte, Fig. S19 (ESI†). This again shows that Fe remains deposited on the Co_3_O_4_ NPs under electrochemical conditions as well as during the OER, in contrast to the Fe dissolution during the OER which has been reported for other Co-oxide catalysts.^25,43^

To track the structural changes in the local atomic environment, extended X-ray absorption fine structure (EXAFS) analysis at the Fe K-edge and Co K-edge was performed, Fig. 4(d), Fig. S16 (ESI†), Co_3_O_4_ spinel structure model,^35,36^ Fig. S20–S22 and Tables S7–S9 (ESI†). Similar to the XANES data, no changes were visible at the Co K-edge with and without Fe decoration due to the low surface-to-volume ratio. At the Fe K-edge, we analyzed the first Fe–O coordination shell and retrieved the Fe–O bond distance which exhibits a reversible contraction during the OER, Fig. 4(e). We have to note that the obtained contraction of the Fe–O distance does not exceed strongly the uncertainty of our fitting. However, in combination with the clear shift of the Fe K-edge, we can conclude that the Fe–O bond contracts reversibly during the OER. Unfortunately, the limited data quality for these Fe-diluted samples does not allow solid quantitative statements on the extent of the Fe–O bond contraction to be provided. Nonetheless, the fact that there is an Fe–O bond contraction suggests the reversible Fe–O oxidation and thus, an accumulation of oxidative charges in the Fe or O ligand environment, as we previously unveiled for the Co–O bond in size-selected 1–5 nm CoO_*x*_(OH)_*y*_ NPs during the OER.^28^ In particular, both the Fe–O bond contraction and the Fe oxidation suggest that Fe sites participate in the redox electrochemistry and thus potentially the OER mechanisms. The Fe–O coordination number was 4.4 ± 0.2 during the conditioning and did not change during the OER. This coordination number is lower than the expected 6 nearest neighbors for octahedrally coordinated, non-spinel, FeO_*x*_ compounds. However, since the XANES spectra still suggest octahedral coordination for Fe species, we attribute this low coordination number to strong structural disorder, resulting in broad, asymmetric bond-length distributions that cannot be treated adequately by simple conventional EXAFS fitting approaches.^28,54,55^ The increase of the Fe–O coordination number after the OER to 5.6 ± 0.3 suggests the integration of the cationic Fe into more ordered structures, such as the Co_3_O_4_ spinel structure, and would match the Fe K pre-edge feature at ∼7112 eV.

**Fig. 5:**
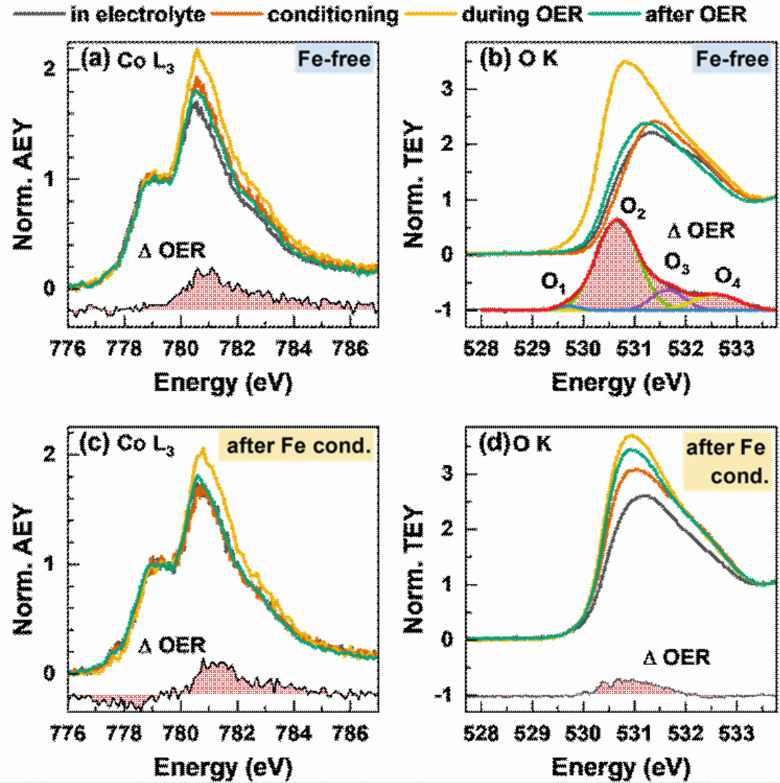
*In situ* near-edge X-ray absorption fine structure (NEXAFS) measurements of Co_3_O_4_ NPs during the OER before (a) and (b) and after (c) and (d) conditioning with 5 ppm Fe. Measurements were performed in 0.1 M KOH with 5 ppm Fe at 1 V_RHE_, after the conditioning at 1 V_RHE_, during the OER at 1.6 V_RHE_ with exchanged, Fe free 0.1 M KOH and after the OER at 1 V_RHE_. (a) and (c) *In situ* Co L-edge Auger electron yield (AEY) spectroscopy (*E*_kin_ = 656 eV) normalized by the intensity at 778.5 eV with the differential spectrum OER – after the OER. (b) and (d) *In situ* total electron yield (TEY) O K spectrum normalized on the intensity at 534 eV of the O K background signal from the membrane and water signal. The differential spectrum OER – after the OER for the Fe-free measurements was fitted with a Gaussian distribution function and shows 4 different oxygen species O_1_–O_2_ created during the formation of electrophilic oxygen.

We further studied the near-surface adaptation using *in situ* Auger electron yield (AEY) near-edge X-ray absorption fine structure (NEXAFS) at the Co L-edge and *in situ* total electron yield (TEY) NEXAFS at the O K-edge, Fig. 5. The Co L_3_-edge spectra show negligible changes between the as-prepared and the conditioned state, but reversible changes during the OER. Here, the Co L_3_-edge spectra were normalized to the feature at 778.5 eV and thus to the amount of tetrahedrally coordinated Co^2+^.^56^

Correspondingly, the peak maximum at 780.5 eV represents octahedrally coordinated Co^3+^ and we can track the near-surface CoO_*x*_(OH)_*y*_ adaption layer that forms reversibly during the OER *via* changes in the Co^3+^ : Co^2+^ ratio. During the OER, the Co^3+^ ratio increases from 1.7 to 2.0 with Fe and 2.2 without Fe conditioning, which suggest a stronger near-surface oxidation in the Fe-free case and that the presence of Fe could suppress the oxidation of the Co sites.

The reversible CoO_*x*_(OH)_*y*_ near-surface and Co^3+*δ*^ formation is also visible through the appearance of a shoulder at 782–783 eV.^28,29,35,56^ In the case of the Fe-decorated Co_3_O_4_, a pre-edge feature at 777.8 eV is more pronounced under non-catalytic conditions, which corresponds to octahedral (high spin) Co^2+^ species. This feature is less strong or absent on the Fe-free Co_3_O_4_ suggesting a lower density of low coordination Co^2+^ sites predominantly present in X-ray amorphous CoO_*x*_(OH)_*y*_.^56^ Interestingly, this feature decreases reversibly during the OER, which in turn suggests a reversible oxidation of the low coordination Co^2+^ sites to Co^3+*δ*^ sites. We note the link between the enhanced OER activity and the presence of these low coordination Co sites in the presence of Fe.

Recent works highlighted the importance of near-surface oxygen chemistry in OER electrocatalysis^27,28,57^ and therefore, we investigated the apparent changes *via in situ* O K-edge NEXAFS, Fig. 5(b) and (d). The features of the pre-edge region at 530–533 eV are caused by octahedrally coordinated Co^3+^/Fe^3+^ μ_3_-O/OH-M^3+^ sites at 531 eV and tetrahedral Co/Fe sites at 532 eV.^56^ The Fe surface decoration leads to a more pronounced feature at 531 eV, which suggests a strong contribution of octahedrally coordinated Fe^3+^ species to the overall signal after conditioning. During the OER, reversible changes in the O K pre-edge region and in the difference spectrum are present for the Fe-free and Fe-decorated Co_3_O_4_. Thus, the reversible increase of the μ_3_-O/OH-M^3+^ signal during the OER is more pronounced under the Fe-free conditions. Notably, the differential spectrum suggests small amounts of electrophilic oxygen species O_1_ at 529.5 eV as well as other species at 530.6, 532 and 533 eV (O_2_ + O_3_ + O_4_) in the Fe-free case.^27,58,59^ In the case of the Fe-conditioned Co_3_O_4_, there is a pronounced contribution of Fe oxide species but interestingly no clear indication for the presence of electrophilic oxygen. Thus, we note that the higher catalytic activity is accompanied by the absence of electrophilic oxygen as well as the presence of redox-active and oxidized Fe^3+^ and high spin Co^2+^ surface species. Also, the lower extent of oxidation from the Co perspective during the OER of the more active Fe-decorated Co_3_O_4_ NP fits to our previous findings in which sub-5 nm CoO_*x*_(OH)_*y*_ NPs oxidize less than expected and exhibit a significantly higher specific activity.^28^

Combining the herein presented results allows us to outline how the surface Fe^3+^ decoration enhances the OER over the crystalline Co_3_O_4_ nanocatalysts. Clearly, the cationic Fe preferably deposits on the (100) facets of the Co_3_O_4_ NPs as compared to low coordination sites of non-cubic Co_3_O_4_ NPs while the surface-averaged Fe : Co ratio saturates at higher Fe coverages (towards 20%). Under these conditions, non-cubic Co_3_O_4_ NP sites are decorated and a slight tendency towards Fe domain formation can be seen. After the OER in the Fe-free electrolyte, the cationic Fe species remain on the surface as shown by XPS, Fig. 3. STEM-EDX analysis suggests that for the low Fe : Co ratios the Fe species appear to redistribute from the (100) facets towards non-cubic Co_3_O_4_, Fig. 3. For these samples, we determined the highest specific activity. This Fe redistribution is in agreement with the concept of dynamic active sites in which *inter alia* cationic Fe species undergo continuous dissolution redeposition cycles within the nanoparticles ensemble. Considering the discrepancy in electrode potential between the conditions of Fe deposition and the OER, our findings suggest that the affinity of Fe deposition on the non-cubic NPs is further enhanced with the increasing electrode potential compared to the cubic NPs. We have to note that any direct proof of the Fe redistribution is challenging and would require additional in-depth electrochemical electron microscopy investigations.

We could correlate the most active surface-averaged Fe : Co ratios with reports in the literature,^17,35,36,60^ although the current increase of the Fe-conditioned crystalline Co_3_O_4_ NPs is not as pronounced as that of the electrodeposited CoO_*x*_(OH)_*y*_ catalysts. This characteristic could result from the lower fraction of non-cubic NPs with a higher density of low coordination sites, which exhibit a lower saturation level for Fe decoration but yielded in the highest specific OER activity. Notably, the obtained Fe decorations are not high enough to obtain decreasing catalytic activities of the Co_3_O_4_ but are stable on the surface even after the OER in the Fe-free electrolyte, contrasting previous reports.^25,43^ We found a clear correlation that a higher Fe surface coverage facilitates the apparent OER activity. In particular, cationic Fe species on Co_3_O_4_(100) facets are beneficial and show a higher saturation level for Fe decoration and thereby overall play a substantial role for the OER activity.^61^ Here, the primary reason for the increased activity is the increase in the electrochemical surface area which can be explained by the overall lower Co_3_O_4_ coherence length and potentially, the stronger structural flexibility of the Co–Fe (oxyhydr)oxide near-surface. Notably, the cationic surface Fe species incorporate into the near-surface spinel lattice which has been reported as the preferred structure under non-catalytic conditions and can be seen by considering the evolution of the Co L-edge and the Fe–O coordination numbers.^35,56^ Supposedly, this recrystallization of Co and Fe sites as a spinel structure is facilitated by the formation of (metal-)MO_*x*_(OH)_*y*_ sites during the OER required as a host to integrate Fe.

**Fig. 6:**
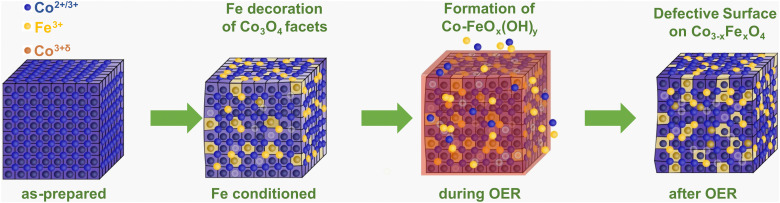
Schematic of the interaction of Fe with cubic Co_3_O_4_ NPs. The incorporation of Fe^3+^ during the conditioning (yellow) leads to a distortion of the Co_3_O_4_ lattice and defect formation (metal vacancies, light blue). During the OER, the formation of the Co–FeO_*x*_(OH)_*y*_ reaction zone (red) comprises a change in the metal oxidation state and metal–oxygen coordination with the dissolution–redeposition cycles shown as Co^2+/3+^ and Fe^3+^ ions near the Co_3_O_4_ NP. After the OER, a more pronounced Fe incorporation into the spinel near-surface as well as defect formation is visible. The O ions are not shown for clarity.

Importantly, our *operando* and *in situ* investigations revealed slight but important differences in the oxygen evolving state of Co_3_O_4_ induced by the presence of near-surface Fe, whose incorporation is schematically shown in Fig. 6. Independent of the Fe decoration, the Co_3_O_4_ NPs oxidize only slightly on the surface as seen in the *operando* surface-sensitive NEXAFS spectroscopy but is also reflected in the contraction of the bulk spinel lattice during the OER identified by *operando* HE-XRD. Without Fe decoration, there was no detectable near-surface structural transformation but in contrast, the near-surface clearly transforms in the presence of Fe as seen in the Co_3_O_4_ coherence length, suggesting the enhanced formation of a Co–Fe (oxyhydr)oxide. Furthermore, the extent of Co–O near-surface oxidation of the more active Fe-decorated Co_3_O_4_ is less pronounced compared to the Fe-free case. While the oxygen near-surface chemistry changes during the OER significantly less strongly, suggesting an apparently lower density of electrophilic oxygen O_1_; this is partially compensated by the oxidation of low coordination Co^2+^ sites and the cationic Fe species during the OER. Thus, we conclude that the presence of low coordination and thus, defect-attributed Co^2+^ as well as redox-active Fe sites lead to a less oxidized state (from the Co and O perspective) during the OER which seems to be linked to the higher catalytic activity. Previously, reducible and/or edge sites in Co(oxyhydr)oxides have been attributed to be crucial (or even responsible) for the OER turnover while exhibiting a lower average Co oxidation state.^28,56^ Similar to the case of NiFe-based OER catalysts, the Fe species seem to decrease the extent of oxidation of the host oxide and increase the density of highly active defect sites. Thus, we conclude that the two-fold influence of the Fe on the OER electrocatalysis over Co-based electrocatalysts is responsible for the OER improvement. At low Fe contents, the specific activity benefits mainly from the electronic interaction between Co and Fe in particular on non-cubic NPs. At higher Fe contents, the Fe incorporation into a Co–Fe (oxyhydr)oxide near-surface leads to a higher active site density dominating the increase in apparent OER activity.

So far, it has been unclear whether the Fe ions in the near-surface of Co- and Ni-based electrocatalysts are redox-active and whether they participate in the OER mechanism. Our findings suggest that the Fe ions in the Co_3−*x*_Fe_*x*_O_4_ near-surface undergo a redox transition and accumulate oxidative charges during the OER. Furthermore, the (homogeneous) incorporation of the Fe ions into the Co_3−*x*_Fe_*x*_O_4_ near-surface as well as the apparent changes in the near-surface electronic structure with a substantially increased conductivity suggest the collective response of the Co, Fe, and O ions on the applied potential. Concurrently, the redox electrochemistry does not suggest pronounced changes in the thermodynamics of the surface oxidation leading to a shift of the barrier for Fe oxidation. Thus, the near-surface Fe ions likely participate in the redox processes during the OER while they improve the catalytic activity.

It is however important to note that the intrinsic heterogeneity of the investigated Co_3_O_4_ electrocatalysts does not allow us to precisely disentangle the catalytic role of *e.g.* the cubic and non-cubic NPs. Recent works suggest that there is a facet dependence of the Co_3_O_4_-catalyzed OER, with a substantially more active CoO_*x*_(OH)_*y*_ near-surface formed on the Co_3_O_4_(100) as compared to that formed on multi-facetted yet crystalline Co_3_O_4_ NPs.^61^ In our case, we focused on revealing trends in the atomic scale interaction between Fe electrolyte species and the CoO_*x*_(OH)_*y*_ near-surface during the OER and their catalytic role. To unambiguously disentangle the catalytic role of cationic Fe species on the Co_3_O_4_ surface, in-depth investigations of model surfaces such as epitaxial Co_3_O_4_ thin films decorated with controlled amounts of Fe are still critically needed. Furthermore, the possible distinct incorporation, distribution and stability of the Fe species on Co oxide particles with different initial structures (*e.g.* cubic *vs.* octahedral or spherical NPs) should be the subject of future studies, if possible, at the single nanoparticle level as demonstrated in the past for other material systems based on particle-on-a-stick experiments.^62–64^

## Conclusions

In this work, we investigated the Fe decoration of Co_3_O_4_ spinel nanoparticles from Fe electrolyte species in detail with *ex situ*, *in situ* and *operando* methods and identified its catalytic role and its influence on the OER active state. We were able to locate the Fe domains predominantly on the (100) facets of cubic NPs. Combining depth-profiling XPS and STEM-EDX, our present findings suggest that Co- and Fe-containing oxide near-surfaces is formed which may result from the formation of CoO_*x*_(OH)_*y*_ during the OER. Notably, we could identify favorable synergies of Fe with non-cubic NPs, although the interaction of Fe with cubic NPs remains the dominant contribution of the OER improvement.

With *in situ* and *operando* methods, we identified both Co and Fe participating in the redox electrochemistry and thus, presumably in the OER although the addition of Fe to the near-surface may hamper the accumulation of Co-related oxidative charges during the OER. Here, we emphasize the lower affinity to electrophilic O-ligands in the presence of Fe accompanied with a higher density of low-coordinated, redox-active Co^3+^ sites and a stronger structural flexibility leading to a more pronounced reaction zone formation. Thus, the near-surface cationic Fe facilitates the OER catalysis in a variety of ways as it irreversibly provides additional reaction centers due to its incorporation into the CoO_*x*_(OH)_*y*_ near-surface during the OER and enhances the OER activity but does not remain solely responsible for the catalysis.

Finally, in this work, we demonstrate how *in situ* and *operando* methods can be used to understand the challenging processes of the formation of active catalyst structures under reaction conditions and extract principles for optimized catalyst design.

## Methods

### Synthesis of Co_3_O_4_ nanoparticles

The synthesis of Co_3_O_4_ was published previously following a solvothermal route.^38^ A mixture of oleylamine and ethanol was added dropwise to a 1 mmol solution of Co(NO_3_)_2_·6H_2_O in EtOH which was stirred rigorously. The mixture was heated in an electrical furnace for 15 h at 180 °C and after precipitation the mixture was centrifuged and washed with EtOH. Finally, the precipitate was dried in air and calcined at 300 °C in air for 1 h. The catalyst ink was prepared with a 1 : 1 mixture of H_2_O and EtOH and no binder like Nafion^©^ was used. The mixture was sonicated for 30 minutes and drop-cast on a glassy carbon electrode.

### X-ray diffractometry

In-house X-ray diffractometry (XRD) was performed with a Bruker D8 Advance diffractometer equipped with a Cu X-ray source in the Bragg–Brentano configuration and variable primary divergence slit. The detector was an energy-dispersive position-sensitive LynxEye XE-T detector (Bruker-AXS). The Rietveld refinement was performed for the Co_3_O_4_ spinel and the CeO_2_ structure taking into account the zero error, sample displacement and the instrumental broadening as determined from the NIST CeO_2_ reference.

### Electrochemistry and catalytic testing for the OER

The OER activity was determined in a three-electrode setup connected to a SP-300 potentiostat (Biologic) and the electrochemistry was performed in a PTFE cell (Pine Research). Prior to each measurement, the electrochemical cell was cleaned with 30% aqueous HNO_3_ solution and rinsed with water to remove metal impurities. Glassware was entirely avoided for all experiments, including *in situ* and *operando* experiments. The catalytic testing was performed in 0.1 M KOH (99.999%, Sigma Aldrich) which was freshly purified with Co(OH)_2_ and checked with inductively coupled plasma mass spectrometry (ICP-MS, iCAP RQ, ThermoFisher Scientific) for metal impurities.^17,65^ If conditioning with Fe preceded the catalytic testing, Fe(NO_3_)_3_·9H_2_O was added from an aqueous stock solution with known concentrations to purify 0.1 M KOH. After conditioning, the electrochemical cell and all contaminated parts were cleaned with nitric acid. Prior to each measurement, the electrolyte was saturated with N_2_. All electrochemical measurements were performed with a rotating disk electrode (RDE, Pine Research) with glassy carbon as the working electrode. The glassy carbon electrode had a diameter of 5 mm with a geometric electrode surface area of 0.196 cm^2^ and was incorporated into a PEEK shaft. The catalyst ink was drop-casted on the electrode tip and dried with Ar for a loading of 50 μg cm^−2^ and the rotation speed was set to 1200 rpm. A graphite rod (Pine Research) with a Zirfon® membrane (Agfa) separated PTFE compartment was used as the counter electrode. The reference electrode was a single junction Hg/HgO electrode (Pine Research) and was calibrated on a daily basis in Fe-free 0.1 M KOH with a commercial reversible hydrogen electrode (HydroFlex®, Gaskatel).

All applied electrochemical potentials are provided *versus* the reversible hydrogen electrode (RHE) and are corrected for the solution resistance which was determined from Nyquist plots from potentio-electrochemical impedance spectroscopy (PEIS) at 1 V_RHE_. The conditioning step of all experiments consisted of 1 h chronoamperometry (CA) at 1.45 V_RHE_ in 0.1 M KOH without Fe or with 0.1, 0.5, 1.0, 3.0 or 5.0 ppm Fe. After the conditioning, the sample was transferred into an Fe-free purified electrolyte for the catalytical testing. The measurements consisted of 2 min CAs from 1.5 to 1.64 V_RHE_ with 20 mV steps and linear sweeps with a 5 mV s^−1^ scan rate from one potential to the next higher one in-between.

For *in situ* and *operando* measurements, the catalytical testing was performed as chronoamperometry at 1.6 V_RHE_ for 30 minutes to provide sufficient scanning times. Measurements in the electrolyte before and after catalysis were usually performed at 1 V_RHE_ if not stated differently.

### Scanning transmission electron microscopy and energy-dispersive X-ray spectroscopy

STEM-EDX was conducted using a ThermoFisher Talos F200x spectrometer at 200 kV. The focused electron beam was scanned over a specific region and EDX spectra were recorded with a 4-quadrant detector (Super-X detection system, ThermoFisher). Thereby, multiple frames were collected to integrate the EDX spectra per frame to enhance the quality of the EDX signal. After the background subtraction, the Fe K and Co K peaks were quantified by Brown-Powell ionization cross sections (Velox 2.13, ThermoFisher Scientific). High resolution STEM images were acquired using a probe-corrected JEM-ARM 200F (JEOL, Japan) equipped with a cold field emission gun (CFEG) operated at 200 kV. The HAADF signal was collected from an electron probe with a 14.2 mrad convergence semi-angle and a 120–430 mrad collection semi-angle. Image acquisition and manipulation were performed with the DigitalMicrograph software v3.43 (Gatan, USA).

### X-ray photoelectron spectroscopy

*Ex situ* X-ray photoelectron spectroscopy (XPS) was performed as-prepared, after conditioning and after catalytic probing. The samples were drop-cast on glassy carbon electrodes.

For laboratory-based XPS measurements, a non-monochromatic Mg anode with 1253.6 eV operated at 250 W was used. The detector was equipped with a hemispherical electron analyzer (Phoibos 100, SPECS GmbH) where a pass energy of 15 eV was applied. The X-ray source and the electron analyzer were aligned in a 54.7° angle.

XPS measurements with constant kinetic energy of photoelectrons were conducted at the ISISS end-station of the BESSY II synchrotron facility of HZB in Berlin. The excitation energy was adjusted for kinetic energies of ∼550 and ∼200 eV. The measured XPS peak areas were quantified with the photon illumination and the photoionization cross-section.^66^ Further information regarding the XPS fitting is provided in Supplementary Note 1 (ESI†).

### *In situ* soft X-ray absorption spectroscopy

*In situ* X-ray absorption spectroscopy (XAS) at the Co L-edge and O K-edge was conducted at the ISISS endstation of the BESSY II synchrotron facility of HZB in Berlin (Proposal No. 222-11340-CR). The samples were spray-coated on FAS-membranes (Fumasep FAS-30, Fumatech) and covered with graphene for conductivity. The AEY signals at the Co L-edge were normalized after the linear background subtraction of the pre-edge region. The O K-edge spectra were corrected with a linear background subtraction and normalization on non-lattice-oxygen features. A SP-300 potentiostat was used for the electrochemical measurements. For an increased stability of the membrane with graphene, the sample was introduced into the vacuum system while flowing the electrolyte and applying 1 V_RHE_ during the pumping process.

### *In situ* high-energy X-ray diffraction

*In situ* high-energy XRD was performed at the ID31 beamline at European Synchrotron Research Facility (ESRF) in Grenoble, France. The photon energy was set to 67 keV and a Dectris Pilatus3 X CdTe 2 M detector was used. The working distance was aligned with a CeO_2_ standard (National Institute of Standards and Technology, Standard Material 674b). The samples were prepared on highly oriented pyrolytic graphite (HOPG) and mounted in a home-made PEEK cell suitable for grazing incidence measurements. A Pt wire was used as the counter electrode and Ag/AgCl (3.4 M KCl, eDAQ ET072) as the reference electrode and all electrodes were connected to a SP-300 potentiostat. Rietveld refinement was performed with the Topas software package.

### *Operando* hard X-ray absorption spectroscopy

*Operando* XAS at the Co and Fe K-edges was carried out at the CryoEXAFS endstation at the KMC-3 beamline of BESSY II synchrotron radiation facility of HZB in Berlin. The XAS measurements were conducted in a self-made single-compartment PEEK cell. The samples were prepared on 10 μm thin glassy carbon electrodes (SIGRADUR®, HTW) and mounted on an orifice of the PEEK cell, fixed and sealed using a Kapton window and penetrable for incoming X-rays and emitted fluorescence signals. The counter electrode was a Pt mesh and the reference electrode was a leak free Ag/AgCl electrode. All measurements were performed in fluorescence mode. The KMC-3 beamline was equipped with a 13-element fluorescence yield detector.

Energy alignment and data normalization were carried out with the software Athena.^67^ Quantitative EXAFS analysis was carried out with conventional least-square fitting as implemented in the FEFFIT8.5 code^68,69^ including complex exchange–correlation Hedin-Lundqvist potential. At the Co K-edge, the first three M–O, Co–M_1_ and Co–M_2_ paths were fitted based on a spinel crystal structure. The amplitudes were calculated following the published procedure and are based on the 2 : 1 ratio of octahedral to tetrahedral sites.^35,36^ At the Fe K-edge, the first M–O path was fitted assuming an octahedral Fe–O coordination. Fitting parameters are provided in Tables S7–S9 (ESI†) and spectra in Fourier transformed *k*-space in note 2 (ESI†).

## Author contributions

FTH, AB and BRC co-wrote the paper. BRC and AB designed and supervised the study. SS performed sample synthesis. FTH designed and performed all electrochemical and catalytic measurements and performed the XPS characterization and data analysis. EO and FPS performed the TEM experiments and data analysis. FTH and AB designed the operando experiments. FTH analyzed the XAS and NEXAFS data. AB analyzed the (HE-)XRD data. FTH, AB, JT, DC, FS, MR, AM, HSJ, AH, UH, and EMD performed synchrotron experiments. All authors contributed to the discussion and editing of the manuscript.

## Conflicts of interest

There are no conflicts to declare.

## Supplementary Material

EE-017-D3EE02809G-s001
