# Supplementary material for: Role of Fe decoration on the oxygen evolving state of Co3O4 nanocatalysts
Source: Energy Environ Sci. 2024 Jan 30;17(5):2046–58. doi: 10.1039/d3ee02809g (PMC10913145; doi:10.1039/d3ee02809g)
Supplement: EE-017-D3EE02809G-s001 [file EE-017-D3EE02809G-s001.pdf]

Electronic Supplementary Material (ESI) for Energy & Environmental Science.
This journal is © The Royal Society of Chemistry 2024

Supplementary Information

Role of Fe Decoration on the Oxygen Evolving State of Co_3O_4 Nanocatalysts

Felix T. Haase[1], Eduardo Ortega[1], Sascha Saddeler[2], Franz-Philipp Schmidt[3], Daniel Cruz[3], Fabian Scholten[2], Martina Rüscher[1], Andrea Martini[1], Hyo Sang Jeon[1], Antonia Herzog[1], Uta Hejral[1], Earl M. Davis[1], Janis Timoshenko[1], Axel Knop-Gericke[3], Thomas Lunkenbein[3], Stephan Schulz[2], Arno Bergmann[1,*], and Beatriz Roldan Cuenya[1*]

[1]Department of Interface Science, Fritz Haber Institute of the Max Planck Society, Berlin, Germany

[2]Institute for Inorganic Chemistry and Center for Nanointegration Duisburg-Essen [CENIDE], University of Duisburg-Essen, Essen, Germany

[3]Department of Inorganic Chemistry, Fritz Haber Institute of the Max Planck Society, Berlin, Germany

e-mail: abergmann@fhi-berlin.mpg.de , roldan@fhi-berlin.mpg.de;

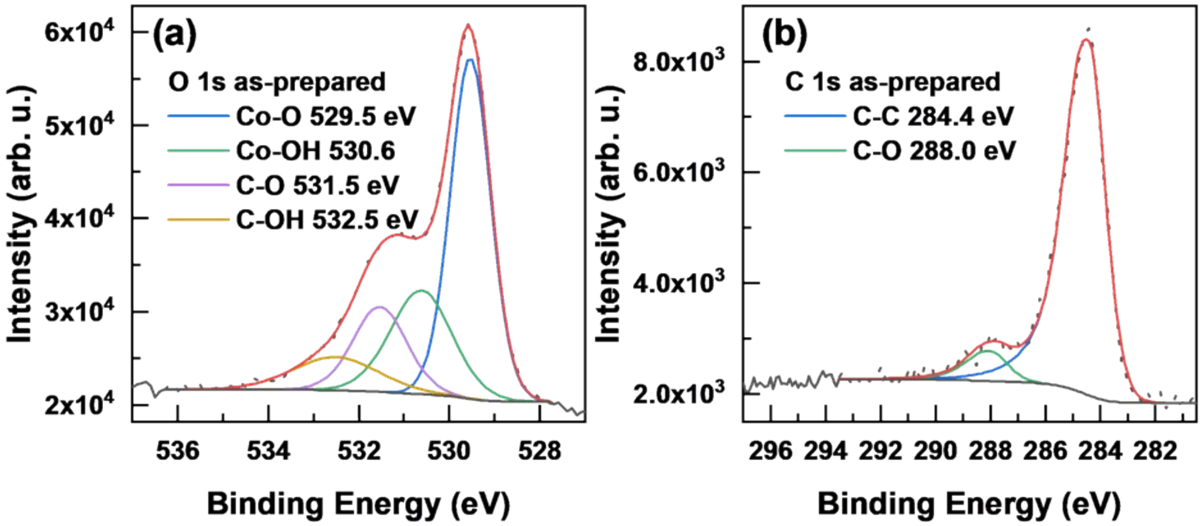

Supplementary Figure 1. X-ray photoelectron spectroscopy of (a) O 1s and (b) C 1s regions of Co_3O_4 nanocatalysts on glassy carbon substrate. The O 1s and C 1s show the absence of organic residues from the synthesis.

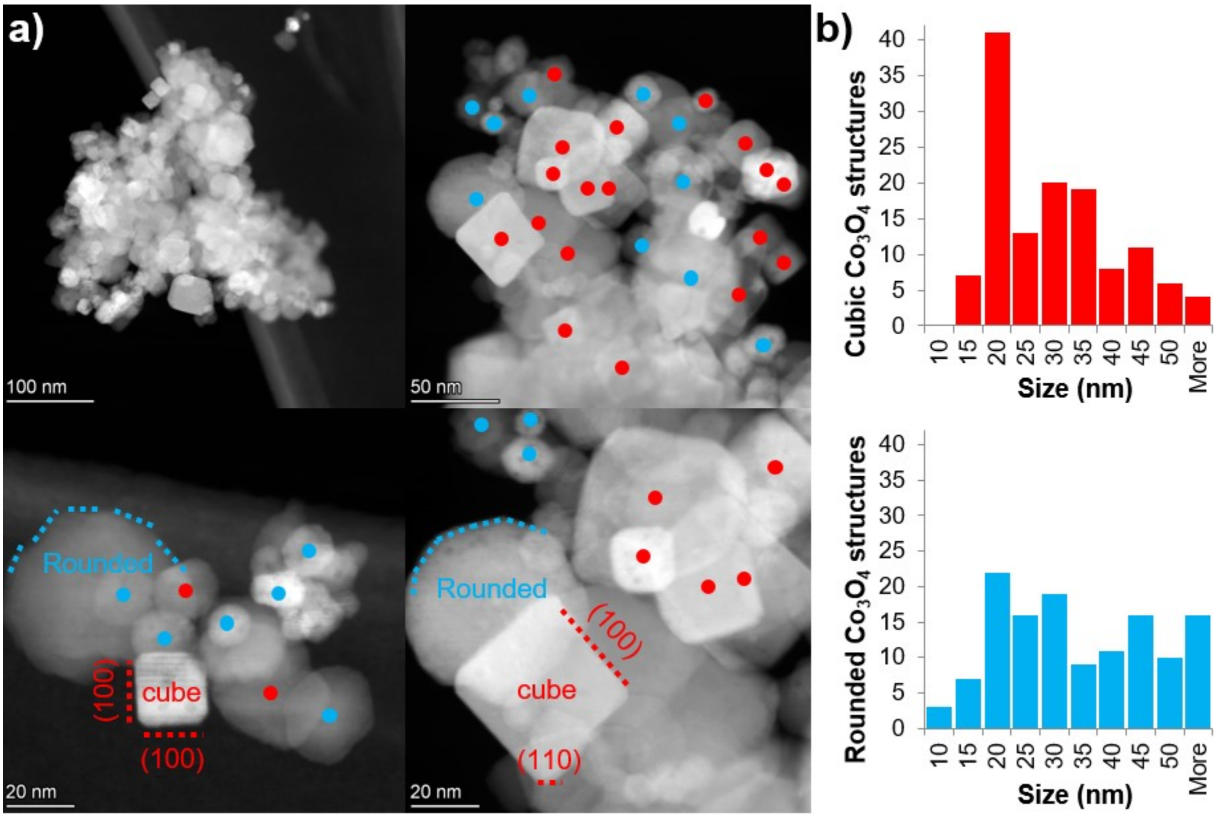

Supplementary Figure 2. STEM images of as prepared Co_3O_4 catalyst at different magnifications (a) to showcase the difference between the highly cubic and the round-shaped particles and (b) their corresponding size-distributions.

Supplementary Table 1: Results from Rietveld refinement of powder XRD data with an added CeO_2 reference (NIST SRM674b). The Rietveld refinement showed two differently sized CeO_2 phases, as well as one Co_3O_4 spinel phase.

	**CeO_2 phase 1**	**CeO_2 phase 2**	**Co_3O_4 phase**
**Space group**	Fm-3m	Fm-3m	Fd-3mZ
**Fraction (wt%)**	19.9	33.9	46.2
**Crystallite size (nm)**	0.95±0.06	314±2	32.8±1.0
**Lattice parameter (Å)**	5.413±0.009	5.4116510	8.08733 ± 0.00011

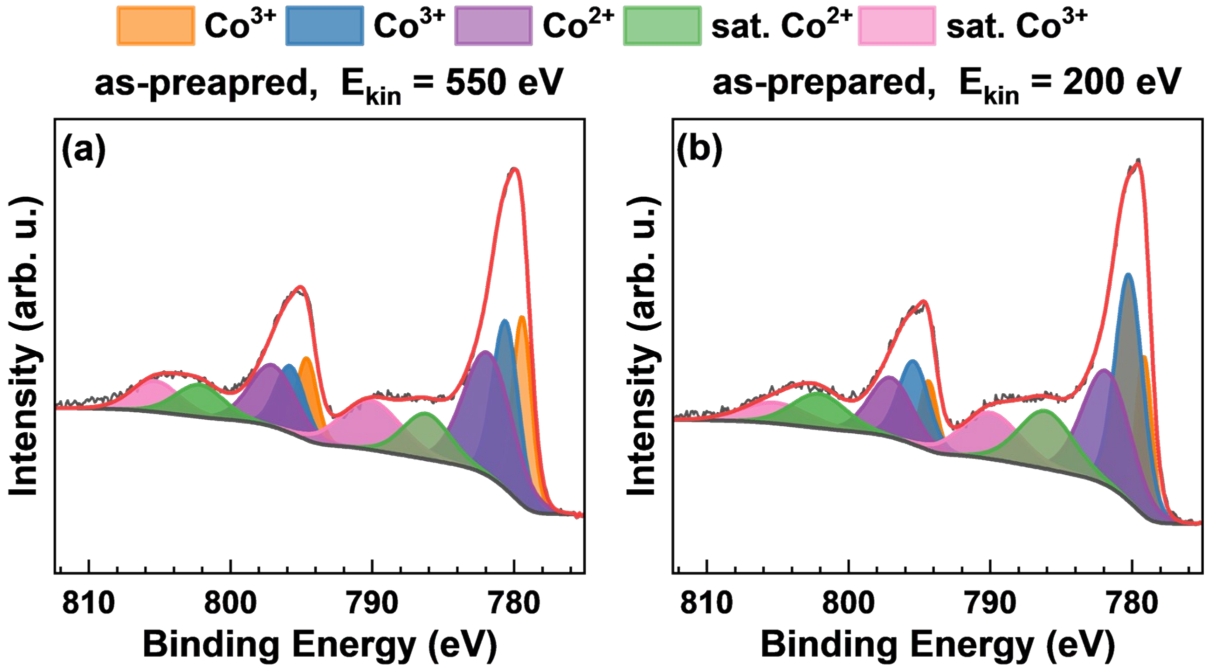

Supplementary Figure 3. Co 2p XPS spectra recorded with different excitation energies to collect photoelectrons with kinetic energies of ~550 and ~200 eV for depth-profiling. Spectra of Co_3O_4 samples in their as-prepared state with (a) ~550 eV and (b) ~200 eV photoelectron energy.[1, 2] The calculated mean free path for ~550 eV kinetic energy is 10.4 Å and 5.6 Å for ~200 eV. Multiplet fitting of Co 2p according to literature.[1, 2]

Supplementary Note 1: XPS fitting Co 2p

All fits for Co 2p are based upon multiplet splitting of Co_3O_4 spinels according to literature.[1, 2] A clear differentiation between Co^{2+} and Co^{3+} in the main peak at 779-782 eV has proven to be challenging due to the strong overlap. However, the satellite features at 786 and 790 eV for Co^{2+} and Co^{3+}, respectively, are clearly assigned based on Co^{2+} references in the literature compared to the Co_3O_4 spinel.[1-4] Thus, the satellite at 786 eV can clearly show Co^{2+} surface enrichment.

Supplementary Table 2: Co 2p XPS parameters of different fitted species as-prepared (Supplementary Figure 3). Provided are binding energies, full-width half maximum (FWHM) and percentage of total peak are for each species.

	Co 2p E_{kin} = 550 eV			Co 2p E_{kin} = 200 eV		
**Species**	**Position (eV)**	**FWHM (eV)**	**Area (%)**	**Position (eV)**	**FWHM (eV)**	**Area (%)**
**Co $2p_{3/2}$**						
**Co^{3+}**	779.4	1.6	12.4	779.2	1.3	8.3
**Co^{3+}**	780.6	2.1	15.1	780.2	2.3	21.3
**Co^{2+}**	781.9	4.0	21.0	781.8	4.0	18.5
**sat. Co^{2+}**	786.2	3.9	7.6	786.1	4.5	10.6
**sat. Co^{3+}**	790.1	5.0	10.5	790.0	4.9	9.2
**Co $2p_{1/2}$**						
**Co^{3+}**	794.6	1.7	5.8	794.4	1.4	3.9
**Co^{3+}**	795.8	2.4	6.8	795.4	2.5	8.5
**Co^{2+}**	797.1	3.8	9.9	797.0	3.6	8.7
**sat. Co^{2+}**	802.2	4.0	5.5	802.1	4.5	6.3
**sat. Co^{3+}**	805.3	4.0	5.3	805.2	5.5	4.6

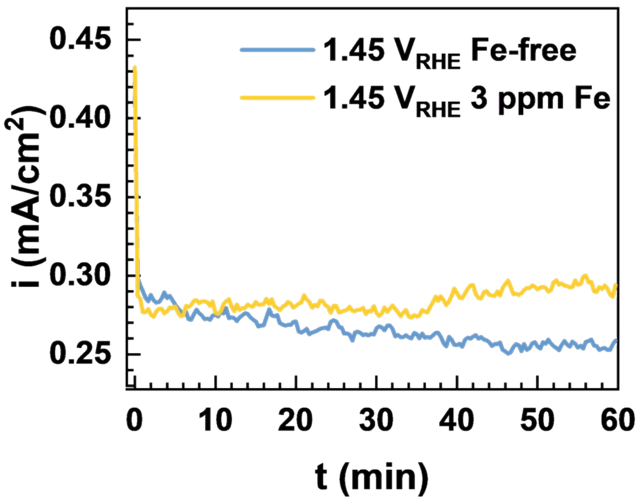

Supplementary Figure 4. Anodic conditioning at 1.45 V_{RHE} in Fe-free and 3 ppm Fe-containing 0.1 M KOH.

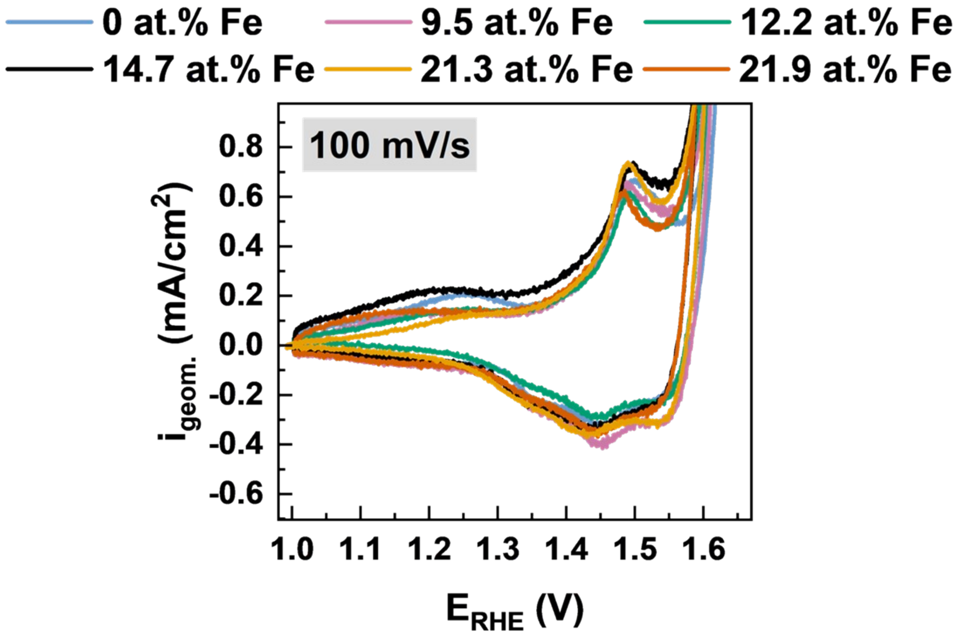

Supplementary Figure 5. Cyclic voltammograms with 100 mV/s scan rate in Fe-free 0.1 M KOH after conditioning.

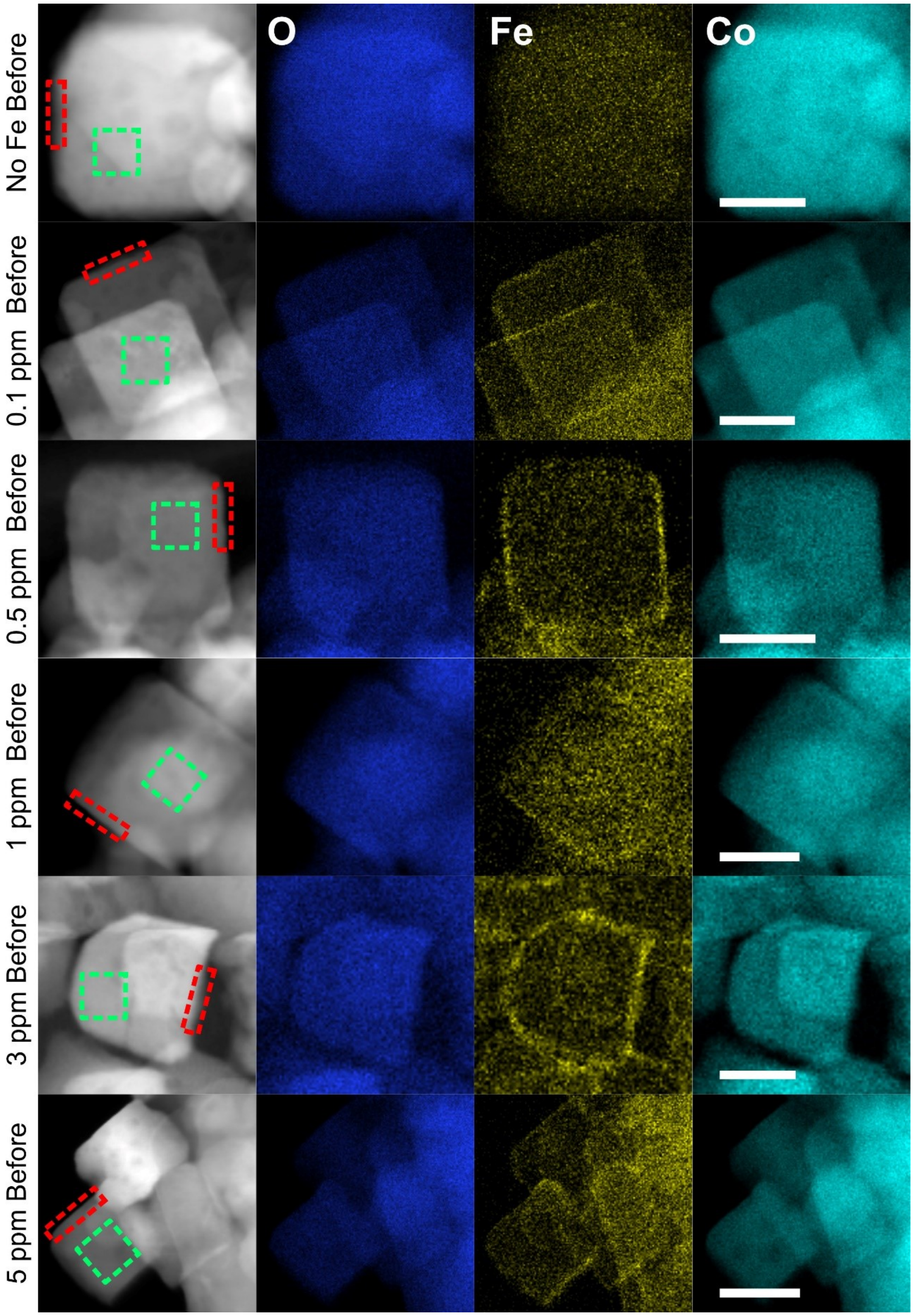

Supplementary Figure 6. STEM-HAADF and corresponding EDX O, Fe and Co-K_α color maps of cubic (low index facets) Co_3O_4 catalysts after conditioning for 1 h at 1.45 V_{RHE} in 0.1 M KOH with different Fe contents. The highlighted regions were used to calculate the atomic percentage values shown in Supplementary Table 2. Scale bar is 20 nm.

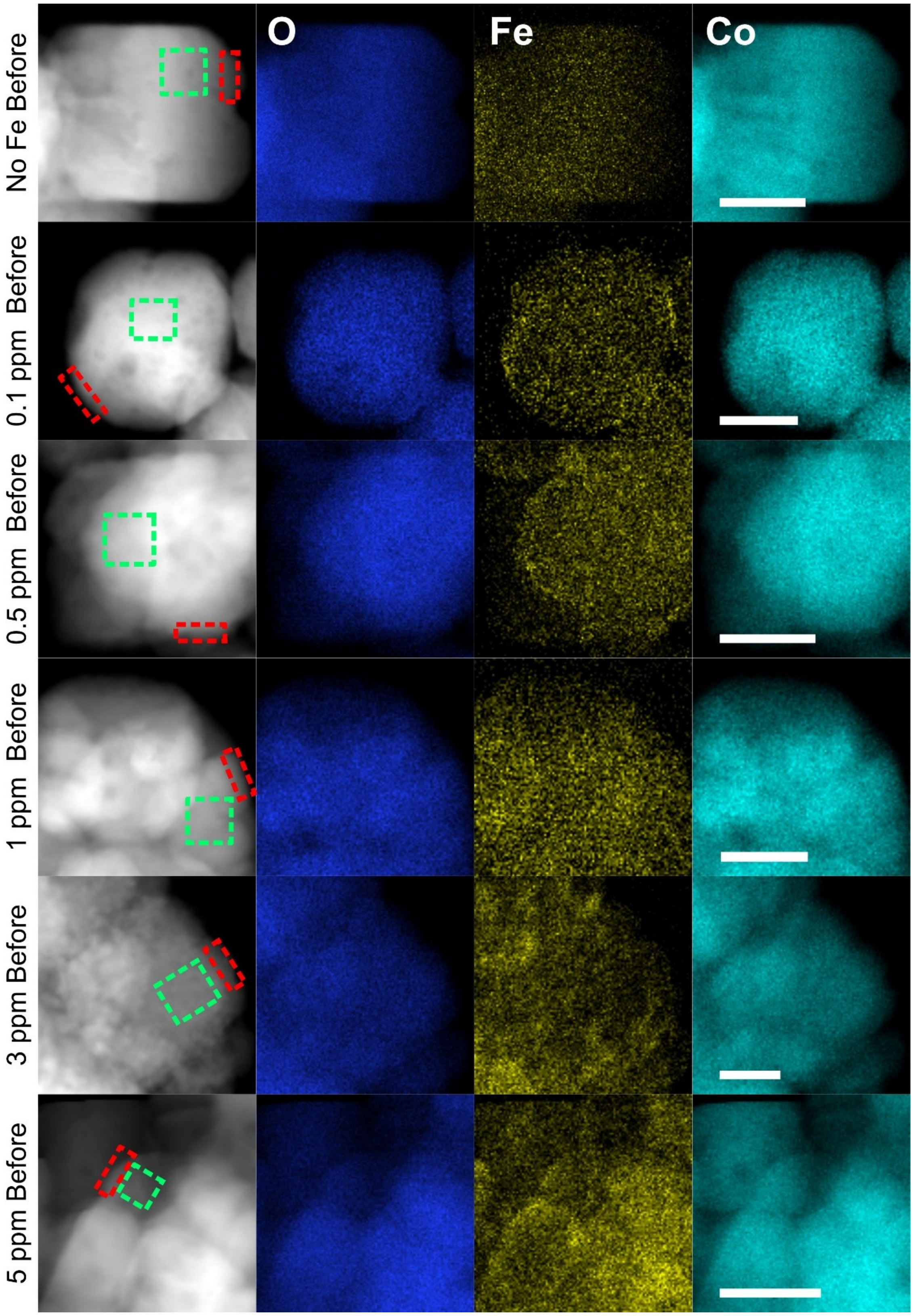

Supplementary Figure 7. STEM-HAADF and corresponding EDX O, Fe and Co-K_α color maps of irregular shape (rounded) Co_3O_4 catalysts after conditioning for 1 h at 1.45 V_{RHE} in 0.1 M KOH with different Fe contents. The highlighted regions were used to calculate the atomic percentage values shown in Supplementary Table 2. Scale bar is 20 nm.

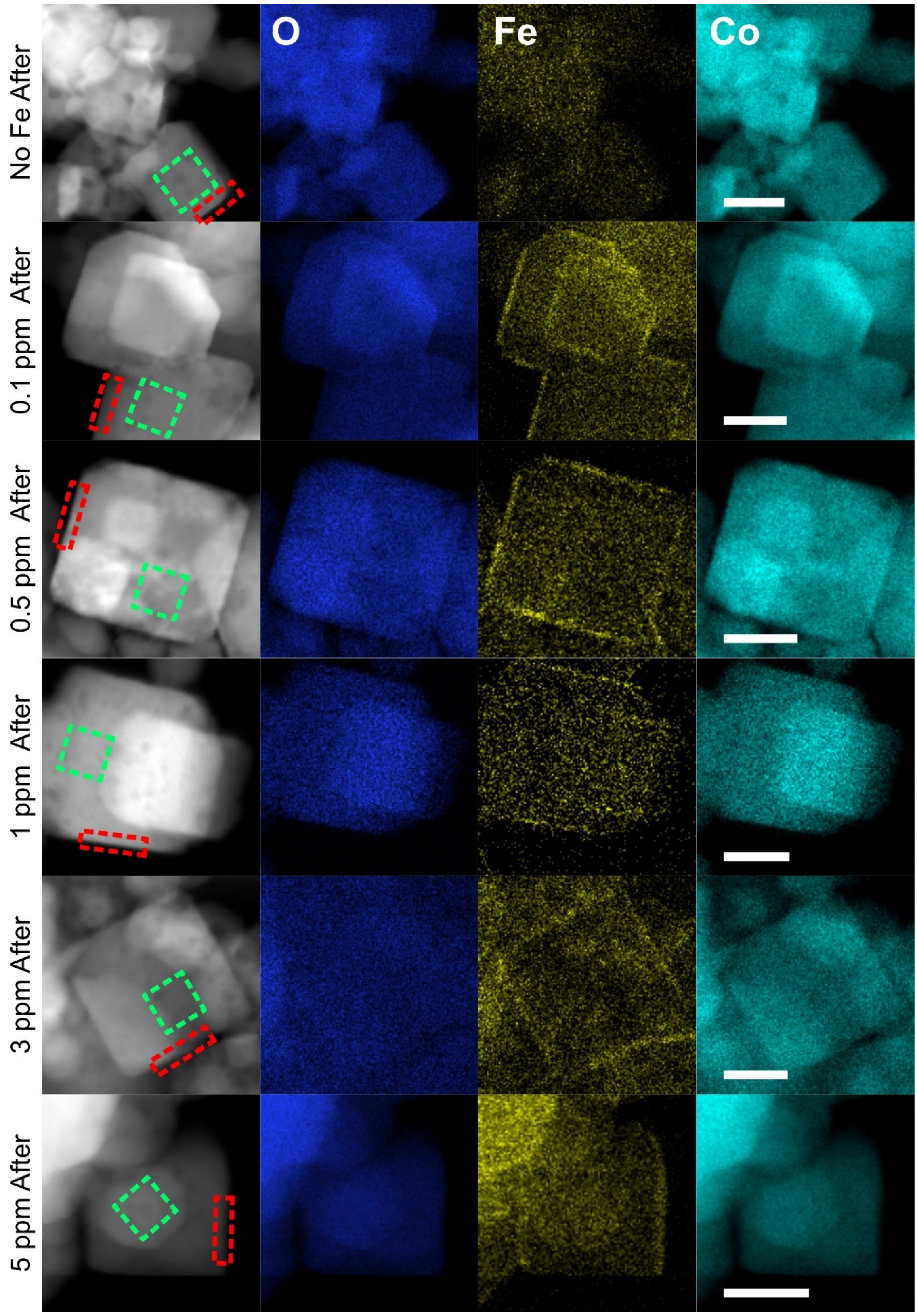

Supplementary Figure 8. STEM-HAADF and corresponding EDX O, Fe and Co-K_α color maps of cubic (low index facets) Co_3O_4 catalysts after potential step experiments. The samples were previously conditioned in a 0.1 M KOH solution with different Fe contents and catalytically propped with potential steps for OER in Fe-free 0.1 M KOH. The highlighted regions were used to calculate the atomic percentage values shown in Supplementary Table 3. Scale bar is 20 nm.

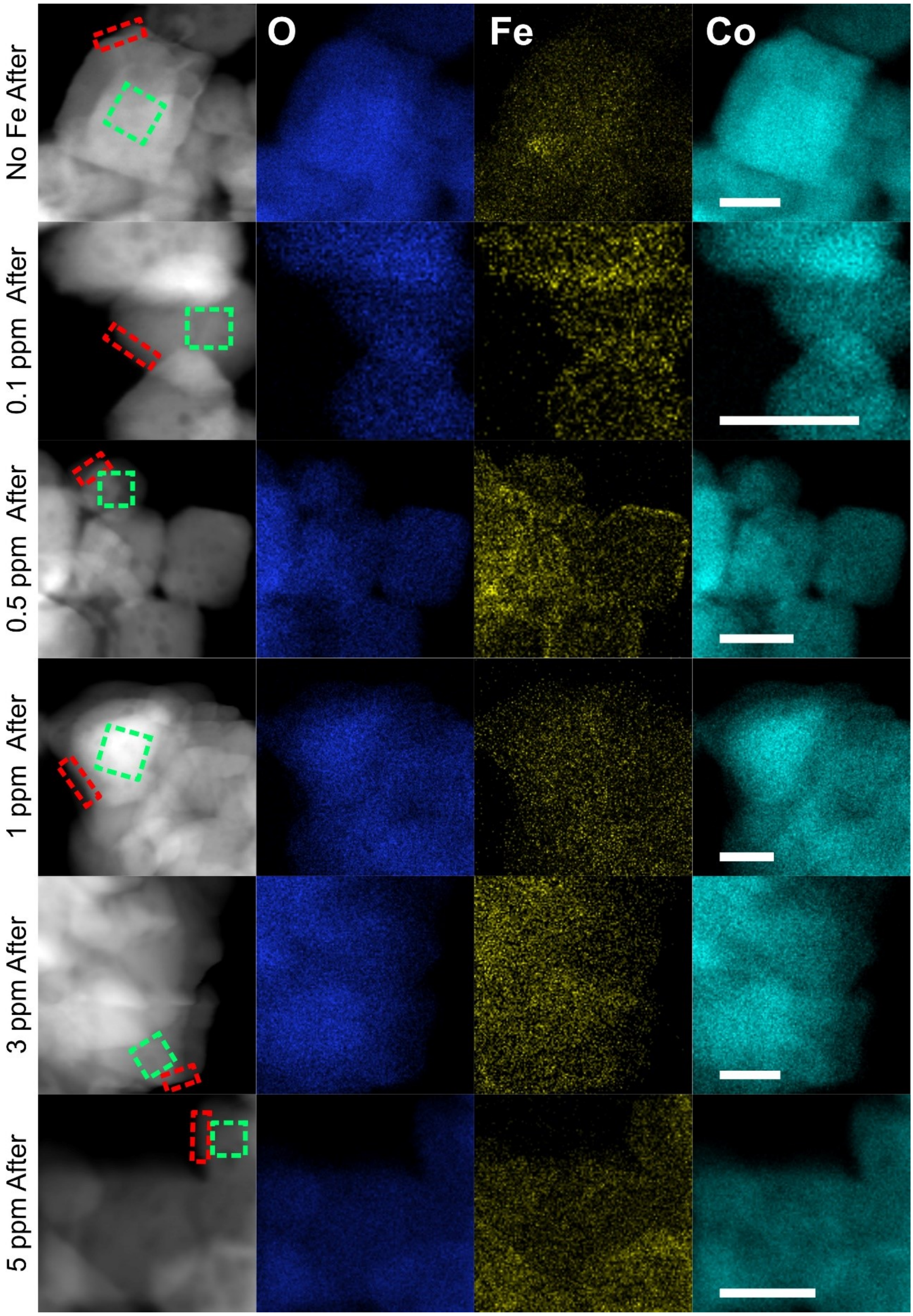

Supplementary Figure 9. STEM-HAADF and corresponding EDX O, Fe and Co-K$_\alpha$ color maps of irregular shape (rounded) Co_3O_4 catalysts after potential step experiments. The samples were previously conditioned in a 0.1 M KOH solution with different Fe contents and catalytically propped with potential steps for OER in Fe-free 0.1 M KOH. The highlighted regions were used to calculate the atomic percentage values shown in Supplementary Table 3. Scale bar is 20 nm.

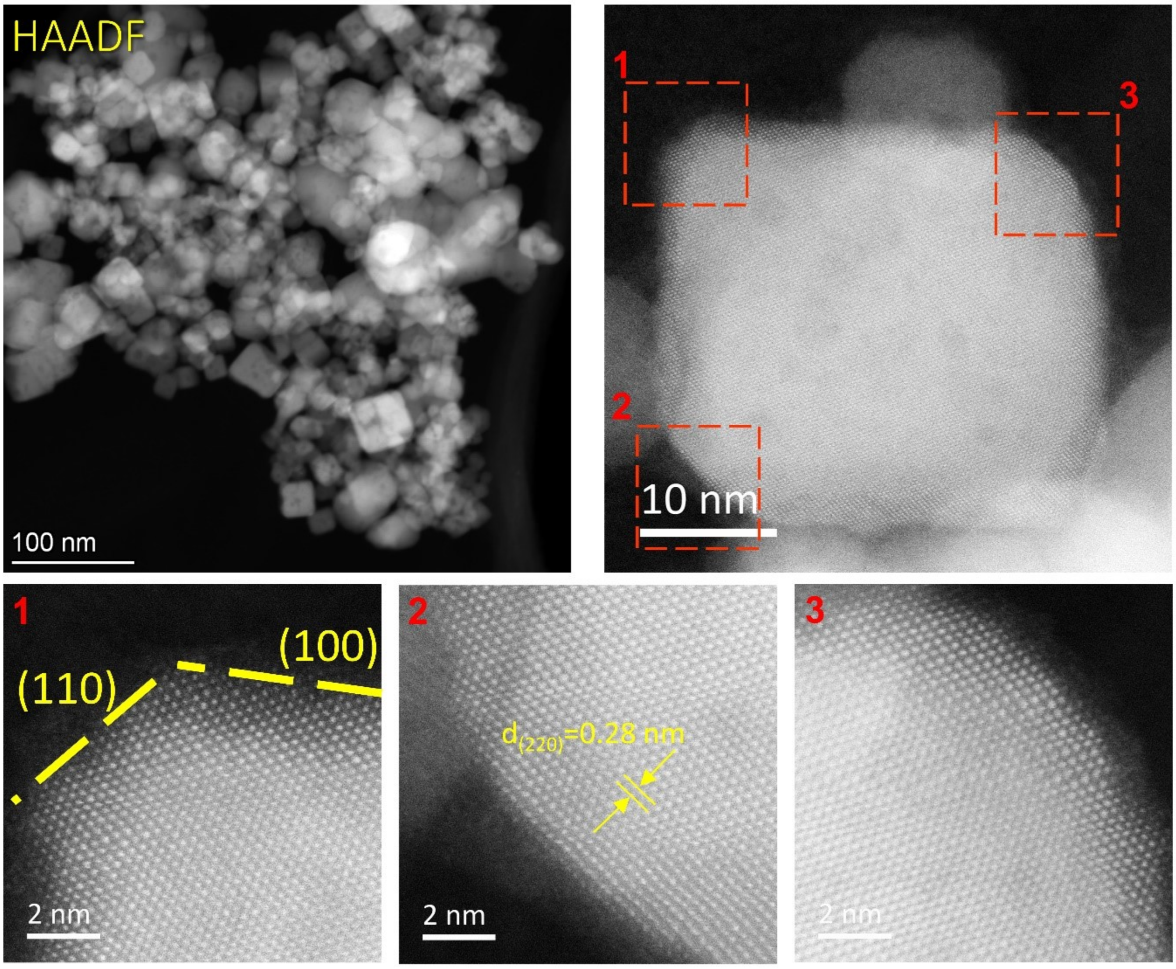

Supplementary Figure 10. High-resolution (HR-) TEM images of Co_3O_4 NPs after conditioning for 1 h at 1.45 V_{RHE} in Fe-free 0.1 M KOH.

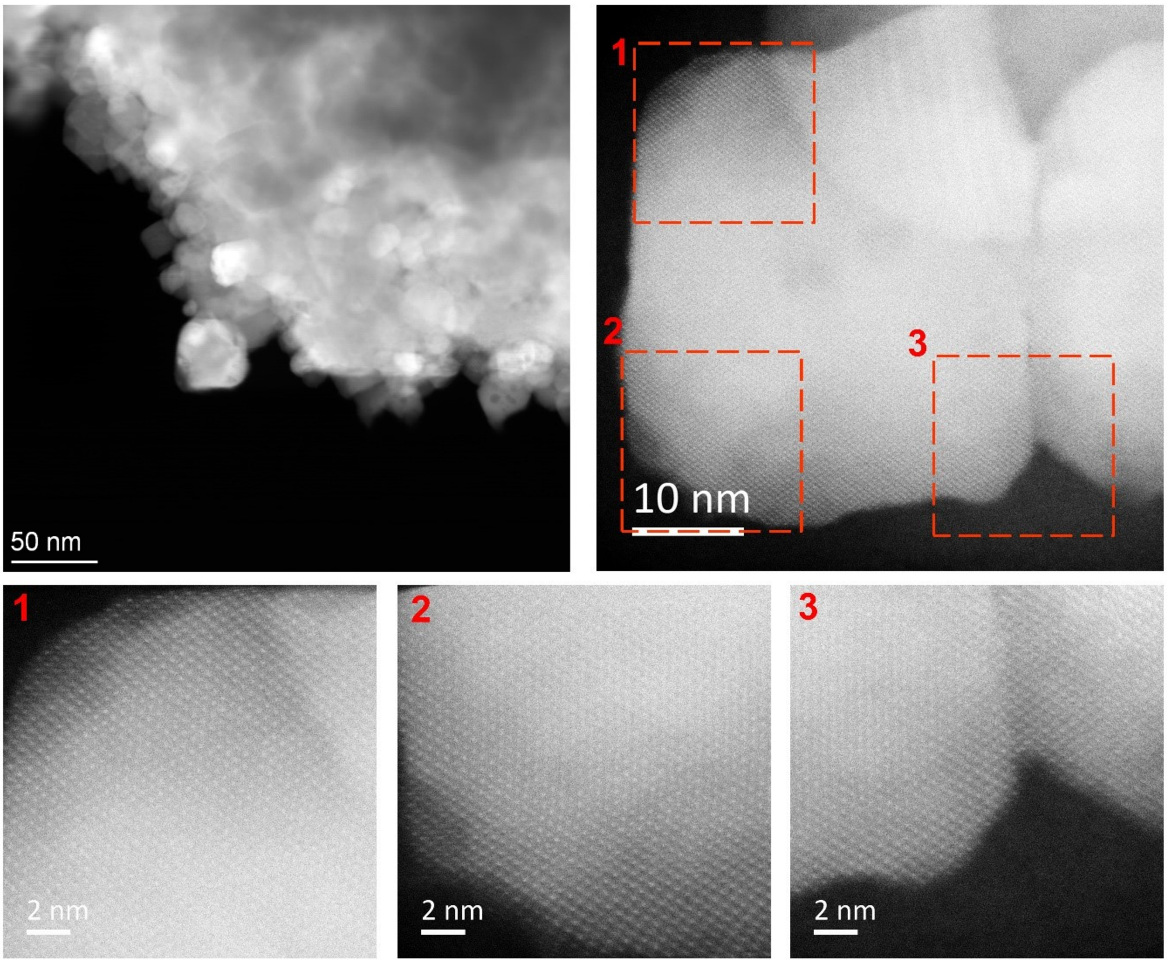

Supplementary Figure 11. High-resolution (HR-) TEM images of Co_3O_4 NPs after conditioning for 1 h at 1.45 V_{RHE} in 5 ppm Fe containing 0.1 M KOH.

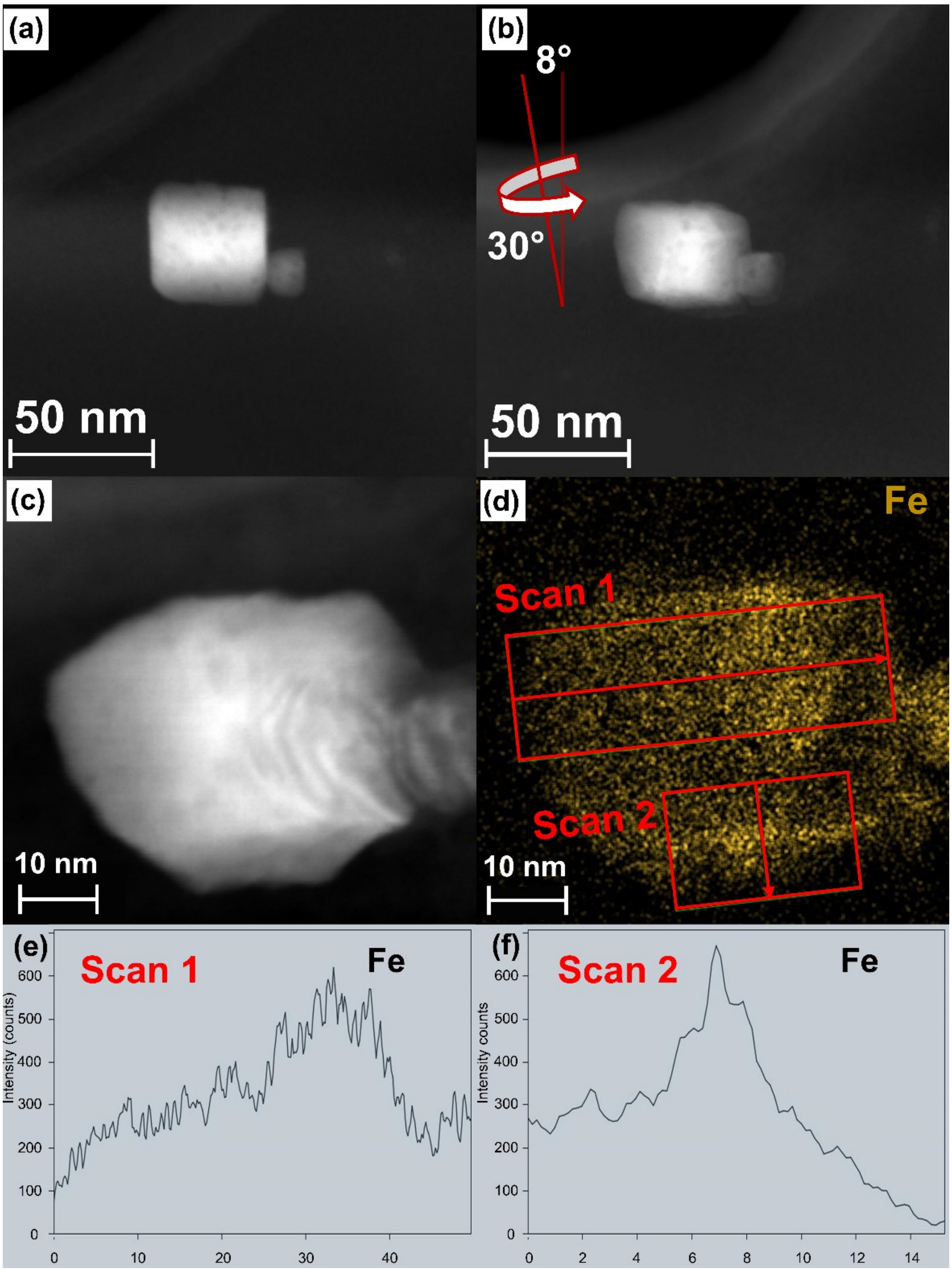

Supplementary Figure 12. STEM-EDX on single cubic Co_3O_4 NPs to locate Fe on faces and edges after conditioning. (a) Leveled and (b) tilted dark-field image of 2 separated nanoparticles. (c) Magnified dark-field image with (d) corresponding STEM-EDX mapping and line scan 1 and 2. Fe counts of line scan 1 (e) and line scan 2 (f) from faces to edges.

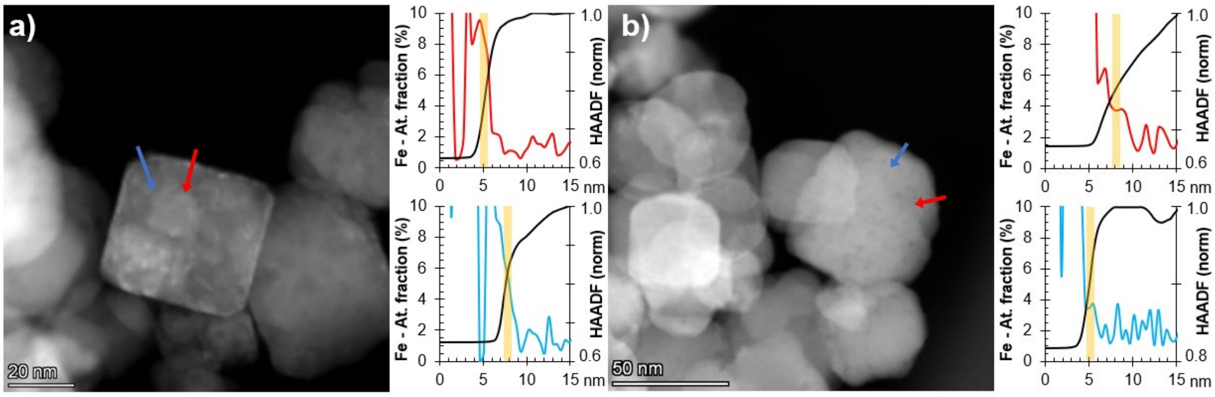

Supplementary Figure 13. STEM-HAADF and corresponding EDX Fe-Atomic fraction (Fe/(Fe+Co) ratio) scan lines from different Co_3O_4 catalysts after conditioning in 0.1 M KOH with 1.0 ppm Fe. The lines represent the regions plotted on the right. (a) The scan line goes through a distinctive cubic structure with (100) and (110) facets (red and blue respectively). (b) A non-cubic nanoparticle has exposed higher order facets with a different Fe-doping profile.

Supplementary Table 3: Fe:Co ratio from background corrected semi-quantitative EDX from X-ray K-lines and XPS measurements after the conditioning in different Fe concentrations both in at.%.

Conditioning		0 ppm	0.1 ppm	0.5 ppm	1.0 ppm	3.0 ppm	5.0 ppm
Cubic NPs (at.%)	(100)	0.6 ± 0.1	3.1 ± 0.5	6.4 ± 0.9	12.3±1.3	16 ± 2	9.3 ± 1.3
	Bulk	0.5 ± 0.1	0.6 ± 0.1	1.5 ± 0.2	1.6 ± 0.2	1.9 ± 0.3	1.5 ± 0.2
Non-cubic NPs (at.%)	Edge	0.5 ± 0.1	2.0 ± 0.5	1.7 ± 0.3	2.6 ± 0.6	8.3 ± 1.2	7.3 ± 1.0
	Bulk	0.5 ± 0.1	0.7 ± 0.1	0.9 ± 0.1	1.4 ± 0.1	2.0 ± 0.3	1.7 ± 0.3
XPS (at.%)		0	9.5	12.2	14.7	21.9	21.3

Supplementary Table 4: Fe:Co ratio from background corrected semi-quantitative EDX from X-ray K-lines and XPS measurements after potential step measurements in Fe-free 0.1 M KOH both in at.%.

Conditioning		0 ppm	0.1 ppm	0.5 ppm	1.0 ppm	3.0 ppm	5.0 ppm
Cubic NPs (at.%)	(100)	0.8 ± 0.2	4.5 ± 0.6	4.4 ± 0.6	7.3 ± 0.8	11.6±1.5	8.7 ± 0.9
	Bulk	0.6 ± 0.1	0.7 ± 0.1	0.8 ± 0.1	1.3 ± 0.2	2.9 ± 0.4	1.1 ± 0.2
Non-cubic NPs (at.%)	Edge	0.7 ± 0.1	2.8 ± 1.0	4.6 ± 0.7	4.2 ± 0.7	3.9 ± 0.7	4.2 ± 0.8
	Bulk	0.5 ± 0.1	1.6 ± 0.3	1.8 ± 0.3	1.6 ± 0.2	2.4 ± 0.4	1.1 ± 0.2
XPS (at.%)		0	8.7	11.8	12.6	20.7	23.4

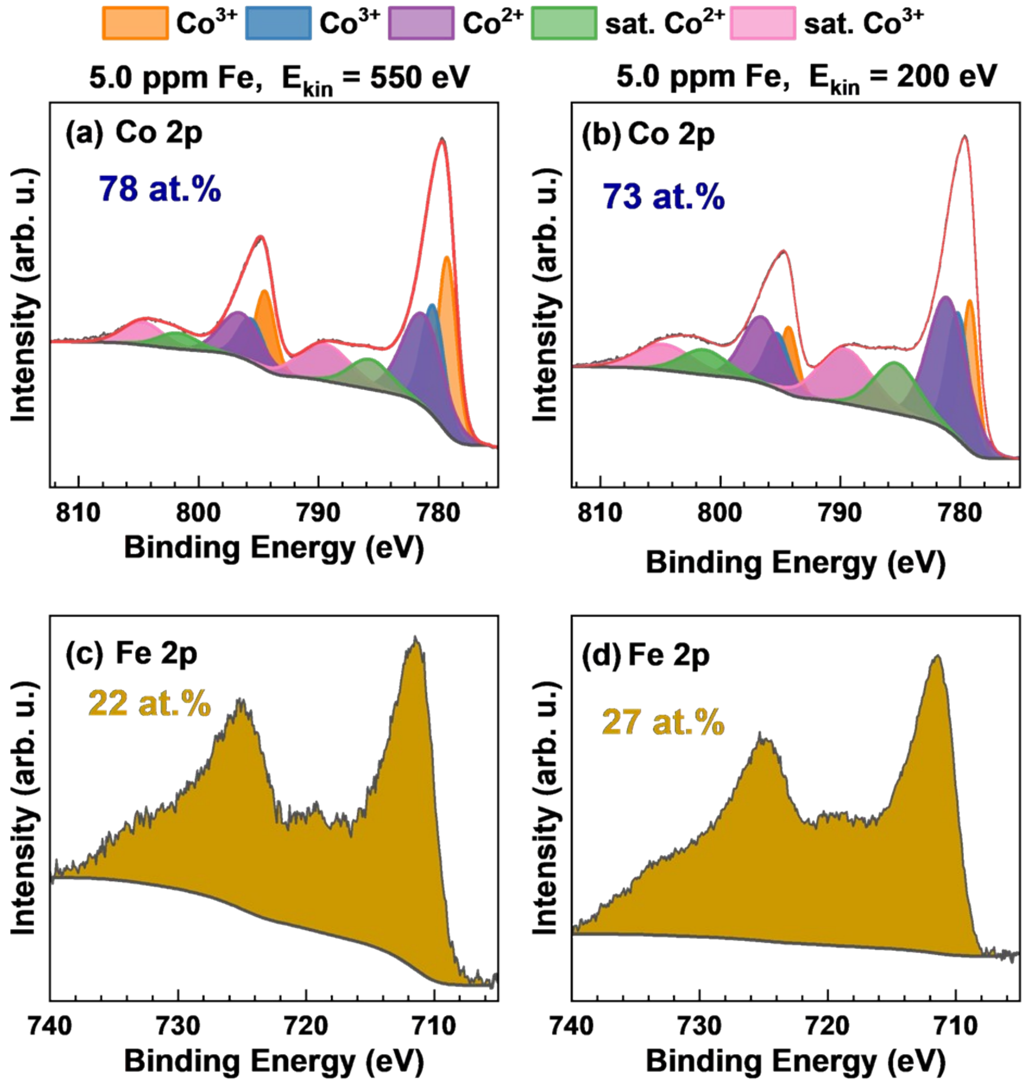

Supplementary Figure 14. Co and Fe 2p XPS spectra recorded with different excitation energies to collect photoelectrons with kinetic energies of 550 and 200 eV for depth-profiling for samples after conditioning in 5.0 ppm Fe containing 0.1 M KOH. The calculated mean free path for 550 eV kinetic energy is 10.4 Å and 5.6 Å for 200 eV. Multiplet fitting of Co 2p according to literature.[1, 2] The Fe 2p XPS clearly show the absence of Fe^{2+} at 709 eV as shown by Diebold et al.[5, 6], thus, suggesting the presence of Fe^{3+} oxide species.

Supplementary Table 5: Co 2p XPS parameters of different fitted species after conditioning (Supplementary Figure 14). Provided are binding energies, FWHM and percentage of total peak are for each species.

	Co 2p E_{kin} = 550 eV			Co 2p E_{kin} = 200 eV		
**Species**	**Position (eV)**	**FWHM**	**area.%**	**Position (eV)**	**FWHM**	**area.%**
**Co 2p$_{3/2}$**						
**Co^{3+}**	779.3	1.8	17.4	779.2	1.5	9.8
**Co^{3+}**	780.5	2.2	13.2	780.2	2.1	11.8
**Co^{2+}**	781.3	4.0	20.4	781.1	3.8	22.6
**sat. Co^{2+}**	785.7	4.0	6.6	785.4	4.3	9.8
**sat. Co^{3+}**	789.5	5.0	10.2	789.6	5.2	12.7
**Co 2p$_{1/2}$**						
**Co^{3+}**	794.5	1.9	8.2	794.3	1.6	4.9
**Co^{3+}**	795.7	2.5	6.0	795.3	2.4	5.9
**Co^{2+}**	796.5	3.9	9.6	796.6	3.8	11.2
**sat. Co^{2+}**	801.7	3.8	3.4	801.4	4.5	5.1
**sat. Co^{3+}**	804.7	4.0	5.1	804.7	5.5	6.3

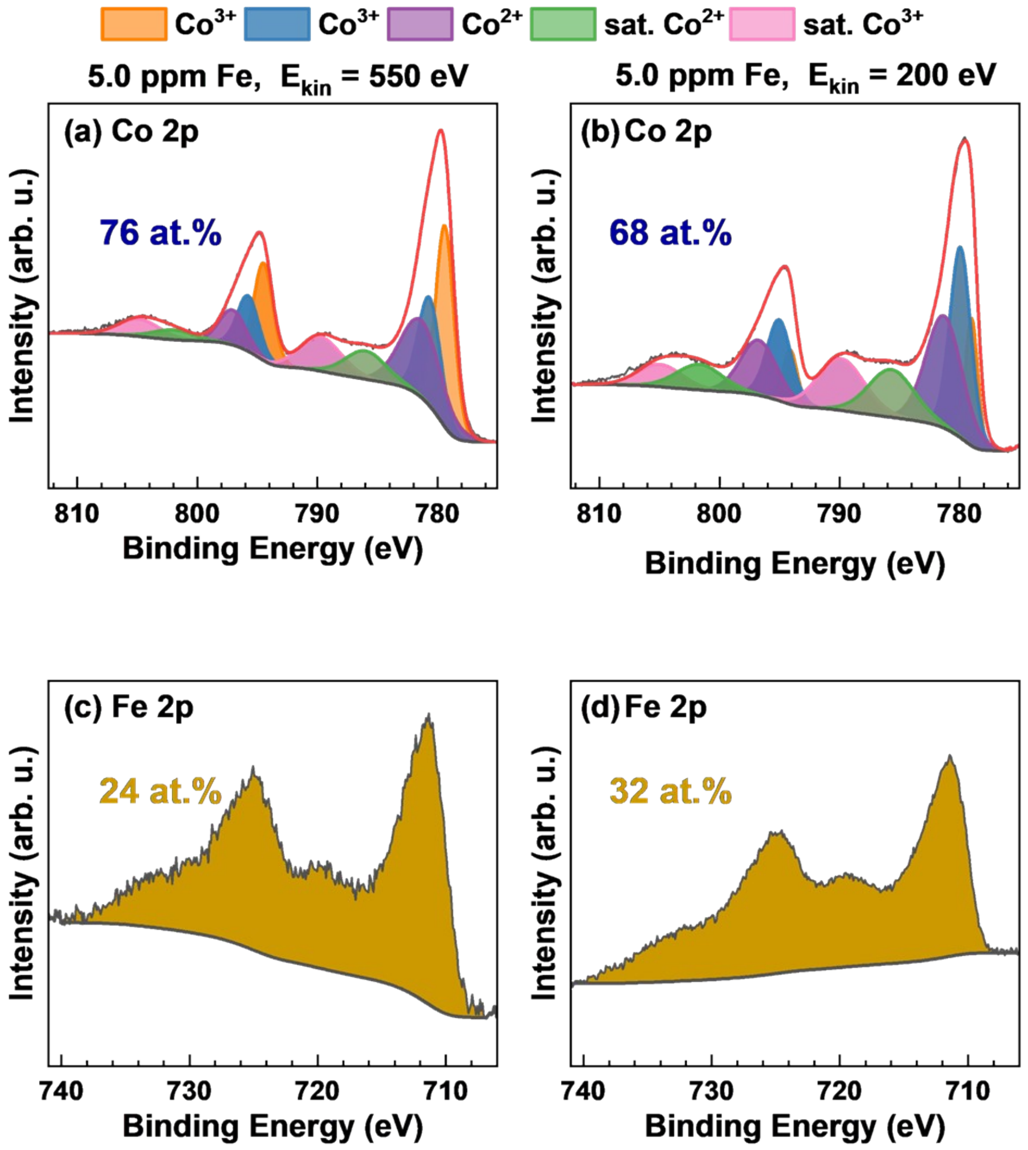

Supplementary Figure 15. Co and Fe 2p XPS spectra recorded with different excitation energies to collect photoelectrons with kinetic energies of 550 and 200 eV for depth-profiling for samples conditioned in 5 ppm Fe after OER steps in Fe-free 0.1 M KOH. The calculated mean free path for 550 eV kinetic energy is 10.4 Å and 5.6 Å for 200 eV. Multiplet fitting of Co 2p according to literature.[1, 2]

Supplementary Table 6: Co 2p XPS parameters of different fitted species after conditioning (Supplementary Figure 15). Provided are binding energies, FWHM and percentage of total peak are for each species.

	**Co 2p E_{kin} = 550 eV**			**Co 2p E_{kin} = 200 eV**		
**Species**	**Position (eV)**	**FWHM**	**area.%**	**Position (eV)**	**FWHM**	**area.%**
**Co $2p_{3/2}$**						
**Co^{3+}**	779.4	1.8	21.7	779.0	1.2	7.4
**Co^{3+}**	780.7	2.2	14.2	779.9	2.1	18.8
**Co^{2+}**	781.4	4.0	18.7	781.3	3.6	19.2
**sat. Co^{2+}**	786.0	4.0	6.9	785.7	4.5	10.4
**sat. Co^{3+}**	789.8	4.1	8.4	789.9	4.5	11.3
**Co $2p_{1/2}$**						
**Co^{3+}**	794.5	1.8	10.8	794.1	1.4	3.7
**Co^{3+}**	795.8	2.1	7.1	795.0	2.4	9.4
**Co^{2+}**	797.1	2.6	5.6	796.8	3.6	9.5
**sat. Co^{2+}**	802.0	4.0	2.4	801.7	4.5	5.6
**sat. Co^{3+}**	804.9	4.0	4.2	805.0	4.3	4.7

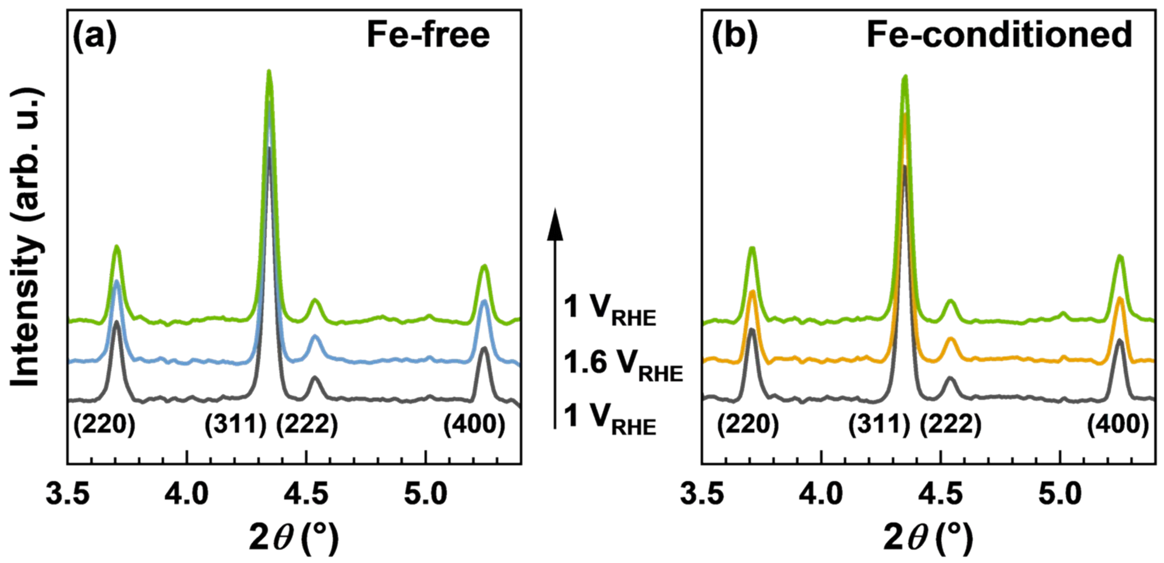

Supplementary Figure 16. Operando high-energy X-ray diffraction pattern excerpts recorded at 67 keV during Fe-free and Fe-conditioned Co_3O_4 with highlighted Bragg reflections of a spinel structure. The electrode potentials are annotated and the sequence of measurements labeled with an arrow.

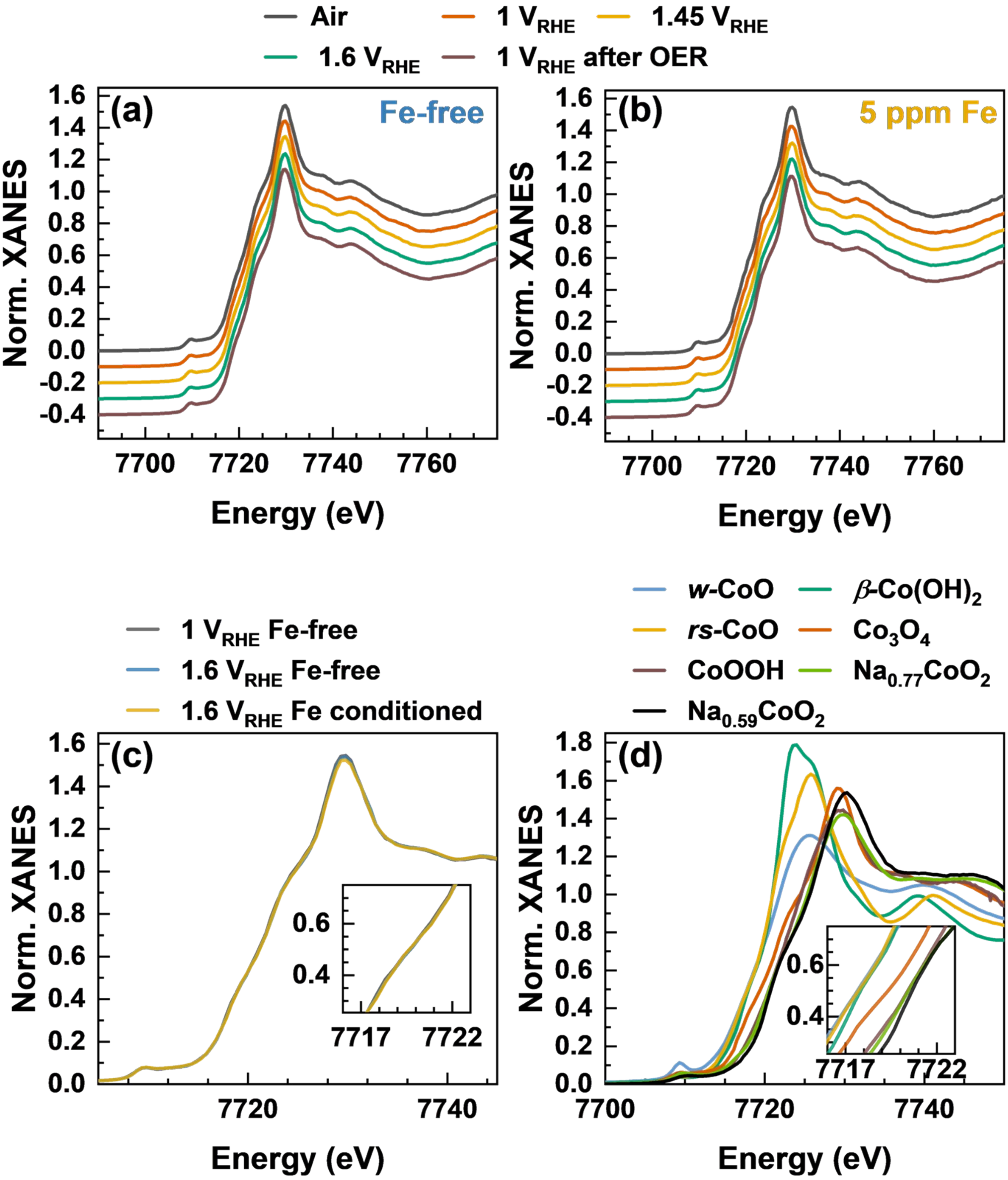

Supplementary Figure 17. (a) Co K-edge XANES after conditioning at 1.45 V_{RHE} in Fe-free 0.1 M KOH. (b) Co K-edge XANES after conditioning at 1.45 V_{RHE} in 5 ppm Fe and subsequent measurements in Fe-free 0.1 M KOH. (c) Comparison of Fe-free and Fe-conditioned XANES during OER at 1.6 V_{RHE}. (d) Co K-edge XANES reference spectra.

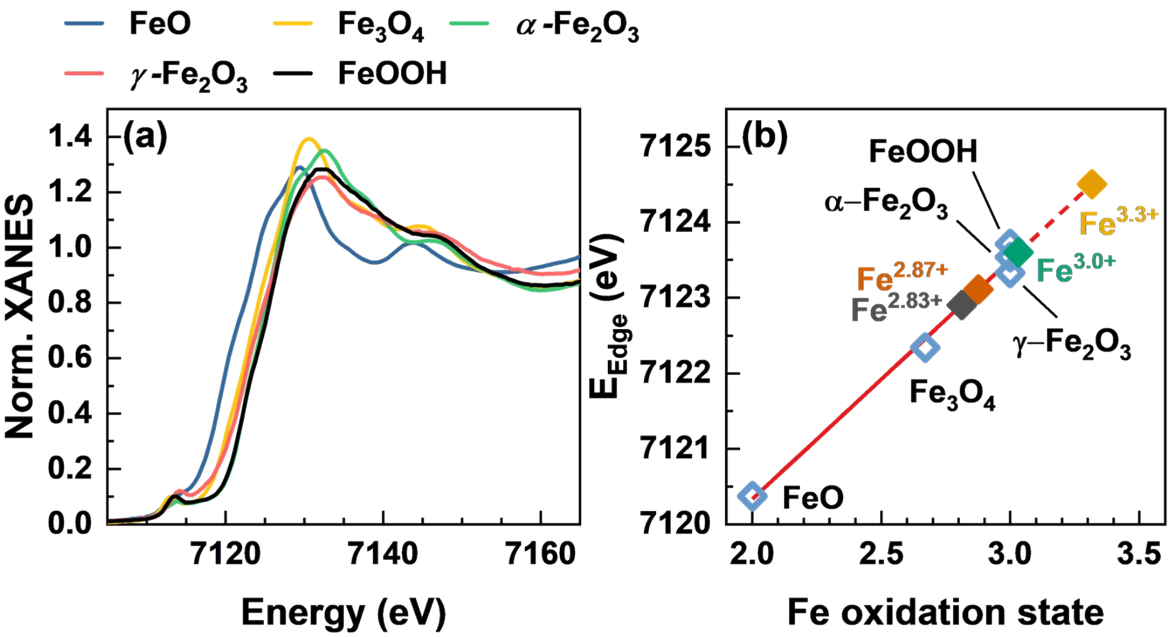

Supplementary Figure 18. (a) Fe K-edge XANES reference spectra and (b) linear calibration for calculation of oxidation states from absorption edge positions of Fe reference materials, displayed in blue.[7, 8]

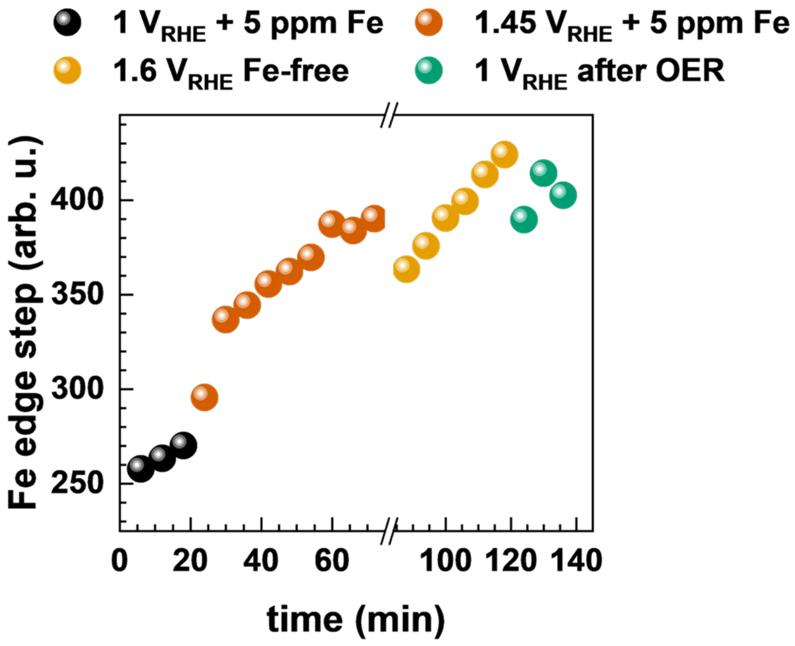

Supplementary Figure 19. Fe K-edge XANES edge step intensity at 1 V_{RHE} in 5 ppm Fe before conditioning, during conditioning at 1.45 V_{RHE} in 5 ppm Fe, during OER at 1.6 V_{RHE} after switching to Fe-free electrolyte and at 1 V_{RHE} after OER in Fe-free 0.1 M KOH.

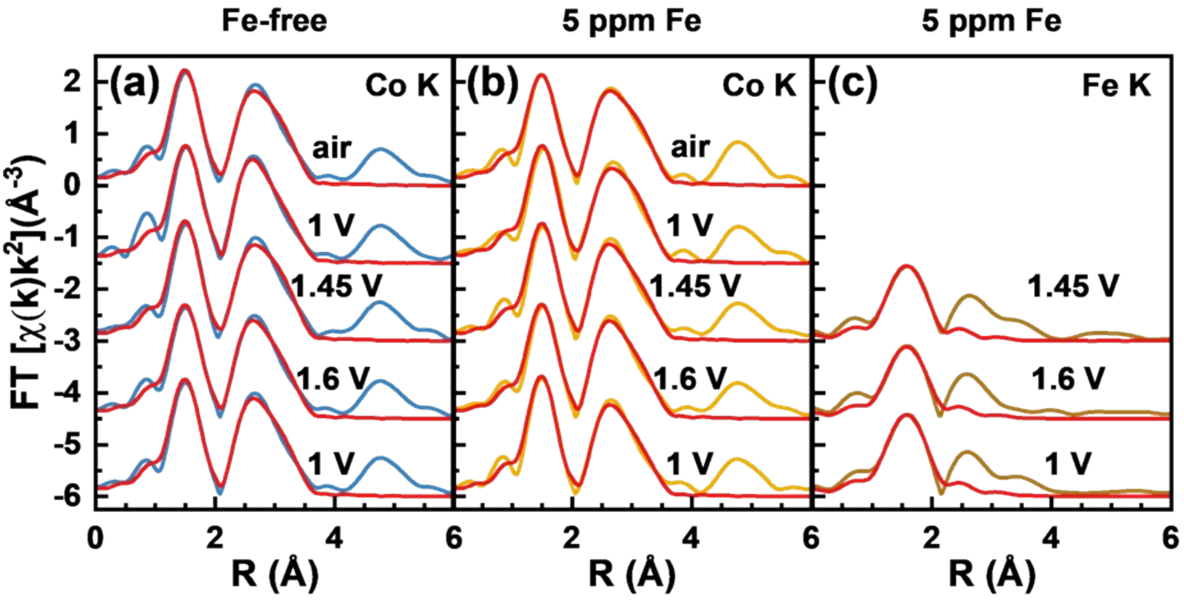

Supplementary Figure 20. Co K-edge FT-EXAFS in R-space with (a) Fe-free and (b), (c) Fe conditioning with 5 ppm Fe in 0.1 M KOH. conditioning.

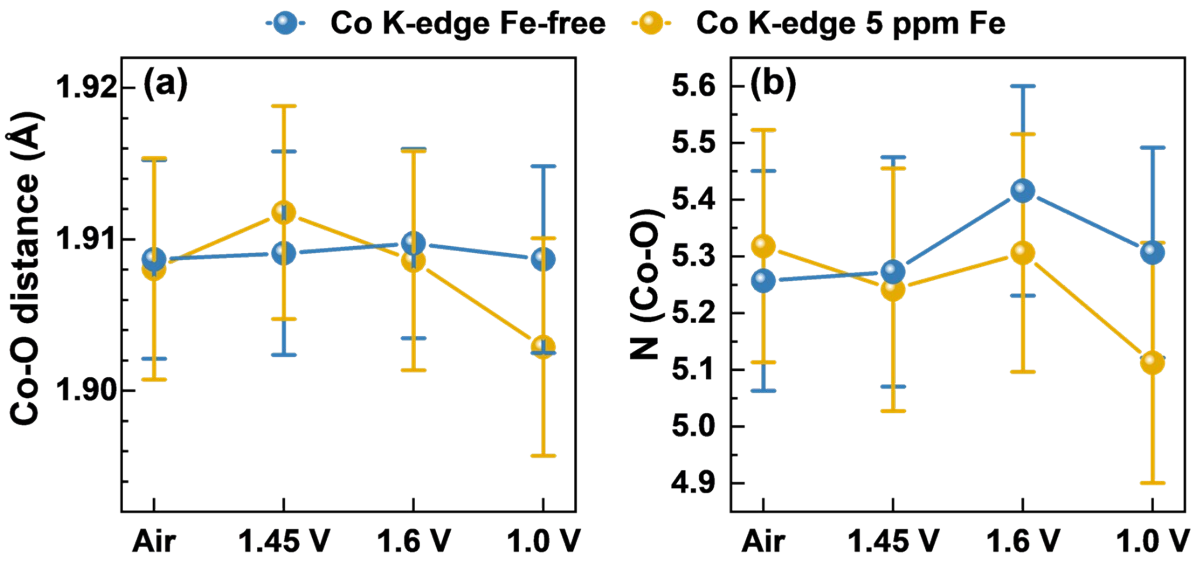

Supplementary Figure 21. (a) Co-O coordination number and (b) Co-O bond distance of the first coordination shell.

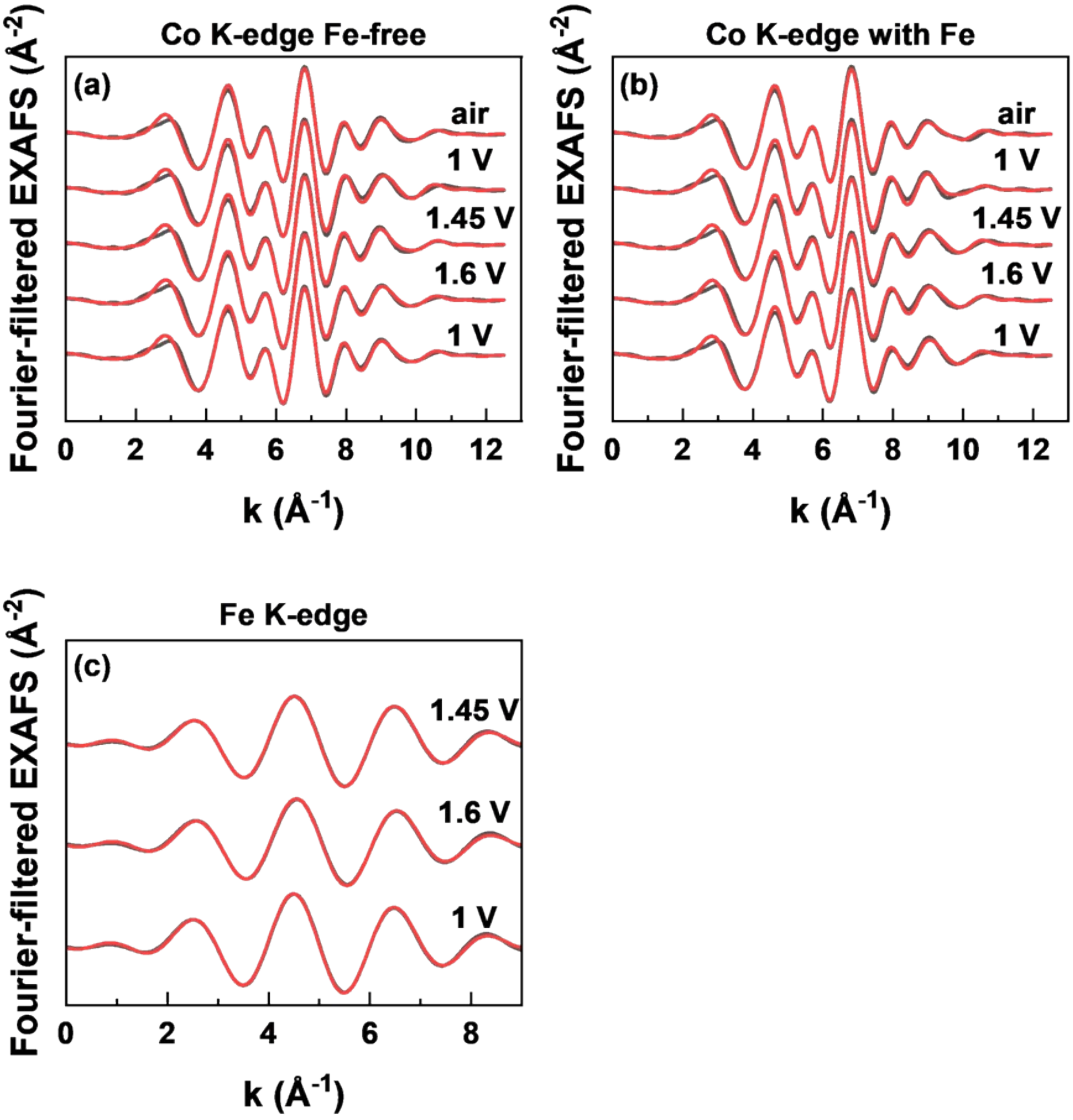

Supplementary Figure 22. Co K-edge FT-EXAFS in k-space with (a) Fe-free and (b), (c) Fe conditioning with 5 ppm Fe in 0.1 M KOH.

Supplementary Note 2: EXAFS fitting

Coordination numbers $\boldsymbol{N}$, interatomic distances $\boldsymbol{R}$, disorder factors $\boldsymbol{\sigma^2}$ and energy shifts $\Delta \boldsymbol{E_0}$ were extracted from EXAFS fitting. At the Co K-edge, the first three coordination shells were fitted and attributed to Co-O, octahedrally oriented Co-M_{Oh} and tetrahedrally oriented Co-M_{Td} paths. Fits at the Fe K-edge include the first coordination shell (Fe-O contribution). All depicted error bars reflect the standard errors calculated from the EXAFS fitting results. At Co K-edge, the coordination numbers for the first 3 paths were calculated using a single variable "x" based on a published routine.[9, 10] The variable x is the fraction of Co in tetrahedral sites from the total number of Co in a spinel phase. The amplitude reduction factors S_0^2 of 0.81 for Co K-edge and 0.7 for Fe K-edge were obtained by fitting EXAFS profiles of Co_3O_4 and α-Fe_2O_3 reference materials with fixed coordination numbers. EXAFS fitting was carried out in R-space in the R-range 1.0-3.8 Å (Co K-edge) or 1.0-2.0 Å (Fe K-edge). At the Co K-edge the k-range used for Fourier transformation was set to 3-10 $Å^{-1}$. For the Fe K-edge, the k-range was set to 3-8 $Å^{-1}$.

Supplementary Table 7: EXAFS fitting parameters with Fe-free 0.1 M KOH conditioned at the Co K-edge.

Fe-free	Co-Path	N	x_{Co}	σ^2 (Å^2)	ΔE_0 (eV)	R (Å)	Fit R-factor (%)
Air	O_1	5.3 ± 0.2	0.3 ± 0.1	0.0038 ± 0.0007	3.2 ± 0.7	1.909 ± 0.007	**2.1**
	M_1	3.9 ± 0.6		0.003 ± 0.001		2.861 ± 0.007	
	M_2	9.3 ± 1.0		0.0073 ± 0.0009		3.367 ± 0.008	
1 V_{RHE}	O_1	5.3 ± 0.2	0.3 ± 0.1	0.0037 ± 0.0009	4.1 ± 0.8	1.920 ± 0.008	**2.7**
	M_1	4.2 ± 0.7		0.002 ± 0.001		2.866 ± 0.008	
	M_2	9 ± 1		0.007 ± 0.001		3.38 ± 0.01	
1.45 V_{RHE} conditioning	O_1	5.3 ± 0.2	0.3 ± 0.1	0.0033 ± 0.0008	3.3 ± 0.7	1.909 ± 0.007	**2.2**
	M_1	4.0 ± 0.6		0.003 ± 0.001		2.860 ± 0.008	
	M_2	9 ± 1		0.0066 ± 0.0009		3.367 ± 0.008	
1.6 V_{RHE}	O_1	5.4 ± 0.2	0.26 ± 0.09	0.0045 ± 0.0007	3.5 ± 0.7	1.910 ± 0.006	**1.8**
	M_1	4.4 ± 0.6		0.004 ± 0.001		2.863 ± 0.007	
	M_2	8.5 ± 0.9		0.0070 ± 0.0009		3.370 ± 0.008	
1 V_{RHE} after OER	O_1	5.3 ± 0.2	0.31 ± 0.09	0.0038 ± 0.0007	3.3 ± 0.7	1.909 ± 0.006	**1.9**
	M_1	4.1 ± 0.6		0.003 ± 0.001		2.861 ± 0.007	
	M_2	9.0 ± 0.9		0.0070 ± 0.0008		3.369 ± 0.008	

Supplementary Table 8: EXAFS fitting parameters with 5 ppm Fe in 0.1 M KOH conditioned at the Co K-edge.

5 ppm Fe	Co-Path	N	x_{Co}	σ^2 (Å^2)	ΔE_0 (eV)	R (Å)	Fit R-factor (%)
Air	O_1	5.3 ± 0.2	0.3 ± 0.1	0.0045 ± 0.0008	3.1 ± 0.7	1.908 ± 0.007	**2.2**
	M_1	4.1 ± 0.6		0.003 ± 0.001		2.856 ± 0.008	
	M_2	9 ± 1		0.0067 ± 0.0009		3.369 ± 0.009	
1 V_{RHE}	O_1	5.3 ± 0.2	0.3 ± 0.1	0.0036 ± 0.0009	3.2 ± 0.8	1.912 ± 0.008	**3.1**
	M_1	4.1 ± 0.6		0.004 ± 0.001		2.861 ± 0.009	
	M_2	9 ± 1		0.007 ± 0.001		3.36 ± 0.01	
1.45 V_{RHE} conditioning	O_1	5.2 ± 0.2	0.3 ± 0.1	0.0035 ± 0.0008	3.5 ± 0.8	1.912 ± 0.007	**2.7**
	M_1	3.9 ± 0.6		0.002 ± 0.001		2.859 ± 0.008	
	M_2	9 ± 1		0.0078 ± 0.0009		3.372 ± 0.009	
1.6 V_{RHE}	O_1	5.3 ± 0.2	0.3 ± 0.1	0.0041 ± 0.0008	3.3 ± 0.8	1.909 ± 0.007	**2.5**
	M_1	4.1 ± 0.6		0.003 ± 0.001		2.859 ± 0.008	
	M_2	9 ± 1		0.007 ± 0.001		3.369 ± 0.009	
1 V_{RHE} after OER	O_1	5.1 ± 0.2	0.4 ± 0.1	0.0029 ± 0.0008	2.7 ± 0.8	1.903 ± 0.007	**2.8**
	M_1	3.5 ± 0.6		0.003 ± 0.001		2.858 ± 0.008	
	M_2	10 ± 1		0.009 ± 0.001		3.36 ± 0.01	

Supplementary Table 9: EXAFS fitting parameters with 5 ppm Fe in 0.1 M KOH conditioned at the Fe K-edge.

**5 ppm Fe**	**Fe-Path**	**N**	**σ^2 (Å^2)**	**ΔE_0 (eV)**	**R (Å)**	**Fit R-factor (%)**
1.45 V_{RHE} conditioning	O_1	4.4 ± 0.2	0.001 ± 0.001	4.2 ± 0.5	1.990 ± 0.005	**0.5**
1.6 V_{RHE}	O_1	4.5 ± 0.3	0.002 ± 0.001	4.8 ± 0.7	1.984 ± 0.007	**0.8**
1 V_{RHE} after OER	O_1	5.6 ± 0.3	0.004 ± 0.001	3.8 ± 0.6	1.994 ± 0.006	**0.6**

References

1. M. C. Biesinger, B. P. Payne, A. P. Grosvenor, L. W. Lau, A. R. Gerson and R. S. C. Smart, *Appl. Surf. Sci.*, 2011, **257**, 2717-2730.
2. R. P. Gupta and S. K. Sen, *Phys. Rev. B*, 1975, **12**, 15-19.
3. F. T. Haase, A. Bergmann, T. E. Jones, J. Timoshenko, A. Herzog, H. S. Jeon, C. Rettenmaier and B. Roldan Cuenya, *Nat. Energy*, 2022, **7**, 765-773.
4. J. Yang, H. Liu, W. N. Martens and R. L. Frost, *J. Phys. Chem. C*, 2010, **114**, 111-119.
5. D. Grumelli, T. Wiegmann, S. Barja, F. Reikowski, F. Maroun, P. Allongue, J. Balajka, G. S. Parkinson, U. Diebold and K. Kern, *Angew. Chem. Int. Ed.*, 2020, **59**, 21904-21908.
6. M. Müllner, M. Riva, F. Kraushofer, M. Schmid, G. S. Parkinson, S. F. Mertens and U. Diebold, *J. Phys. Chem. C*, 2018, **123**, 8304-8311.
7. J. Dittmer, L. Iuzzolino, W. Dörner, H.-F. Nolting, W. Meyer-Klaucke and H. Dau, *Photosynthesis: Mechanisms and Effects*, 1998, **2**, 1339-1342.
8. H. Dau, P. Liebisch and M. Haumann, *Anal. Bioanal. Chem.*, 2003, **376**, 562-583.
9. S. Saddeler, G. Bendt, S. Salamon, F. T. Haase, J. Landers, J. Timoshenko, C. Rettenmaier, H. S. Jeon, A. Bergmann, H. Wende, B. Roldan Cuenya and S. Schulz, *J. Mater. Chem. A*, 2021, **9**, 25381-25390.
10. F. T. Haase, A. Rabe, F.-P. Schmidt, A. Herzog, H. S. Jeon, W. Frandsen, P. V. Narangoda, I. Spanos, K. Friedel Ortega, J. Timoshenko, T. Lunkenbein, M. Behrens, A. Bergmann, R. Schlögl and B. Roldan Cuenya, *J. Am. Chem. Soc.*, 2022, **144**, 12007-12019.
